# Neural network models of autonomous adaptive intelligence and artificial general intelligence: how our brains learn large language models and their meanings

**DOI:** 10.3389/fnsys.2025.1630151

**Published:** 2025-07-30

**Authors:** Stephen Grossberg

**Affiliations:** Departments of Mathematics and Statistics, Psychological and Brain Sciences, and Biomedical Engineering, Boston University, Boston, MA, United States

**Keywords:** neural network, ChatSOME, learning, recognition, cognition, language, emotion, consciousness

## Abstract

This article describes a biological neural network model that explains how humans learn to understand large language models and their meanings. This kind of learning typically occurs when a student learns from a teacher about events that they experience together. Multiple types of self-organizing brain processes are involved, including content-addressable memory; conscious visual perception; joint attention; object learning, categorization, and cognition; conscious recognition; cognitive working memory; cognitive planning; neural-symbolic computing; emotion; cognitive-emotional interactions and reinforcement learning; volition; and goal-oriented actions. The article advances earlier results showing how small language models are learned that have perceptual and affective meanings. The current article explains how humans, and neural network models thereof, learn to consciously see and recognize an unlimited number of visual scenes. Then, bi-directional associative links can be learned and stably remembered between these scenes, the emotions that they evoke, and the descriptive language utterances associated with them. Adaptive resonance theory circuits control model learning and self-stabilizing memory. These human capabilities are not found in AI models such as ChatGPT. The current model is called ChatSOME, where SOME abbreviates Self-Organizing MEaning. The article summarizes neural network highlights since the 1950s and leading models, including adaptive resonance, deep learning, LLMs, and transformers.

## Learning language meanings from viewing visual scenes

### Neural network models that can realize artificial general intelligence

This article continues to develop a neural network model of the key brain processes that enable a child or adult to learn language utterances and their meanings. Learning language meanings includes learning of associative links between language utterances and the perceptual events in the world that they describe, as well as the learner's emotional responses to these events. Such learning typically begins when a baby who knows no language listens to, interacts with, and imitates people who do, often parents or other caregivers.

Grossberg (2023) modeled how multiple brain regions interact to support the initial learning of language utterances and their meanings. This explanation is built upon biological neural network models of how our brains make our minds, which have been incrementally developed over the past half century. These models provide principled and unifying explanations of data from many psychological and neurobiological experiments about essentially all the main processes, whereby our brains make our conscious minds in both healthy individuals and clinical patients. These include models of how we consciously see, hear, feel, and know things about the world, and use our conscious states to effectively plan and act to realize valued goals.

The brain processes that are modeled to enable these explanations include vision and visual object recognition; audition, speech, and language; development; attentive learning and memory; cognitive information processing and social cognition; reinforcement learning and motivation; cognitive-emotional interactions, including reinforcement learning; navigation; cognitive and motor planning; sensory-motor control and robotics; and mental disorders, such as Alzheimer's disease, autism, medial temporal amnesia, schizophrenia, ADHD, PTSD, auditory and visual agnosia and neglect, and disorders of slow wave sleep. These models involve many parts of the brain, ranging from perception to action, and multiple levels of brain organization, ranging from individual spikes and their synchronization to cognition. The models have also been applied and specialized to solve large-scale problems in engineering, technology, and AI.

Taken together, these models provide a blueprint for what I call Autonomous Adaptive Intelligence, or AAI, while others may prefer the term Artificial General Intelligence, or AGI. By either name, these models may be realized by neural network models and architectures, as well as by physical embodiments in the controllers of many types of machines, including VLSI chips and adaptive mobile robots. A self-contained and non-technical overview and synthesis of this progress over the past 50+ years is described by Grossberg (2021b). All the articles by Grossberg et al. cited in this article can be downloaded from https://sites.bu.edu/steveg.

The analysis in Grossberg (2023) was restricted to the learning of short language utterances and their perceptual and affective meanings. Typical sentences were “Watch mommy throw the ball” or “Look at mommy throw the ball.” These sentences and their meanings were explained in terms of how learned brain representations of the sentences, and their associative links to learned brain representations of perceptual and affective experiences, are learned in real time as a child interacts with a teacher.

To realize AAI or AGI, this kind of language competence needs to be generalized to the learning of large numbers of language utterances and their perceptual and affective meanings. This is the goal of the current article. This article explains that two parallel streams of research activity in AI have developed over the years, with little interaction between them. This article will hopefully help correct that problem.

This enhanced neural network model provides an alternative to the large language models, or LLMs, like ChatGPT, that some AI practitioners believe can provide a foundation for AGI. This belief is not supported by the well-known fact that LLMs, due to the way in which they promiscuously heap together information that they take from the internet, have no values, intelligence, or goals, and literally do not know what they are talking about.

For example, a 2025 article entitled *Proof or Bluff: Evaluating LLMs on 2025 USA Math Olympiad* (Petrov et al., 2025) concluded that “Using expert human annotators, we evaluated several state-of-the-art reasoning models on the six problems from the 2025 USAMO within hours of their release. Our results reveal that all tested models struggled significantly, achieving <5% on average.”

In addition, the deep learning models that are used to help create LLMs are *untrustworthy* (because they are *not explainable*) and *unreliable* (because they can experience *catastrophic forgetting* of their learned memories at any time while being trained using hundreds or thousands of trials by slow off-line learning). These are just two of the 17 serious computational problems that have long been known about the back propagation model that is the learning algorithm used by deep learning and that were never faced by the other research stream in AI (Grossberg, 1988, 2020). Despite these problems, deep learning and LLMs have recently been used in many applications, notably by Google DeepMind. Partly, this is due to the advent of huge databases on the internet (e.g., pictures of cats) and networks of extremely fast and powerful computer servers. It is primarily due to the fact that many models help to solve *model-independent problems* that any reasonable model can handle. The future of AAI and AGI will depend, I contend, on models that have avoided the computational problems noted above, such as the ones summarized in this article.

As in Grossberg (2023), to contrast the present work with ChatGPT, I call my model the ChatSOME model, where the abbreviation SOME stands for Self-Organized MEaning.

To extend ChatSOME to incorporate large language corpora, neural network models are needed of brain processes whose emergent properties give rise to the following kinds of psychological functions, over and beyond those used in Grossberg (2023). This extension is possible because all these models have already been published in archival articles, which include principled explanations and quantitative computer simulations of large amounts of psychological and neurobiological data that validate their concepts, neural mechanisms, and emergent properties.

The current article will not repeat technical details that have been published in these articles. Rather, I will provide heuristic explanations to make the article self-contained, along with citations of the original archival articles for readers who want to know details.

The main scenario that the article models is one in which a learner explores arbitrarily many visual scenes while a teacher guides the learner's visual attention to different parts of the scene and uses language to describe what the learner is seeing.

This competence requires an analysis of how a young learner's brain may achieve visual scene understanding even before learning how to describe parts of the scene using language.

After linguistic descriptions of many scenes are achieved with the help of teachers, a child or adult can explore new scenes using previously learned skills to understand them even without an explicit teacher.

Several brain processes are needed to achieve such competencies. Only those that are essential for a heuristic understanding will be described so that the article does not become too long.

How our brains achieve visual *figure-ground perception* of the objects in a 3D scene, a 2D picture, or a screen. Separating objects from each other and from their backgrounds in scenes or screens is needed before individual objects can be attended, learned, and recognized. This process was modeled in articles such as Grossberg (1993, 1994, 1997, 1998, 2016), Grossberg and McLoughlin (1997), Grossberg and Pessoa (1998), Grossberg and Wyse (1991, 1992), and Kelly and Grossberg (2000).

How our brains use *binocular fusion* of our two eyes to enable *eye movements to scan a 3D scene* while *learning invariant recognition categories* of the objects in the scene. Binocular fusion is the process by which the scenic images received by each of our two eyes are fused into a single image that is perceived in depth. Invariant object recognition enables recognition of an object from any of its views, positions, and sizes. These processes were modeled in articles such as Cao et al. (2011), Fazl et al. (2009), and Grossberg et al. (2014).

How our brains achieve *scene understanding* by using scenic context to efficiently drive a *visual search* that shifts *spatial attention* and eye movements around the scene while *incrementally learning the scene's objects and object positions, as well as the spatial contexts within which they occur in the scene*. These processes were modeled in articles such as Cao et al. (2011), Fazl et al. (2009), Foley et al. (2012), Gancarz and Grossberg (1999), Grossberg and Huang (2009), Grossberg et al. (1997, 2012, 2014), Huang and Grossberg (2010), Silver et al. (2011), and Srihasam et al. (2009).

These abilities are combined in a more comprehensive neural network model of how our brains solve the *Where's Waldo problem*; namely, how perceptual, cognitive, and emotional brain processes cooperate during learning to categorize and find desired objects in a cluttered scene. These processes were modeled in the study by Chang et al. (2014).

These abilities are possible because our brains can consciously see, hear, feel, and know things about the world that they experience, and use their conscious states to plan and act to realize valued goals. The discovery of how, where in our brains, and why, from a deep computational perspective, humans experience conscious states arose from a sustained analysis over many years of *how humans learn quickly without experiencing catastrophic forgetting*; that is, how we solve the *stability-plasticity dilemma*. Adaptive resonance theory, or ART, has explained this in a series of articles starting with Grossberg (1976a,b, 1978a, 1980) and culminating in articles and books such as Grossberg (2017, 2018, 2019b, 2021a,b, 2025).

ART is the most advanced cognitive and neural theory that explains how humans learn to attend, recognize, and predict objects and events in a changing world that is filled with unexpected events. ART explained and simulated data from hundreds of psychological and neurobiological experiments, and also made confirmed predictions.

**Figure 1:**
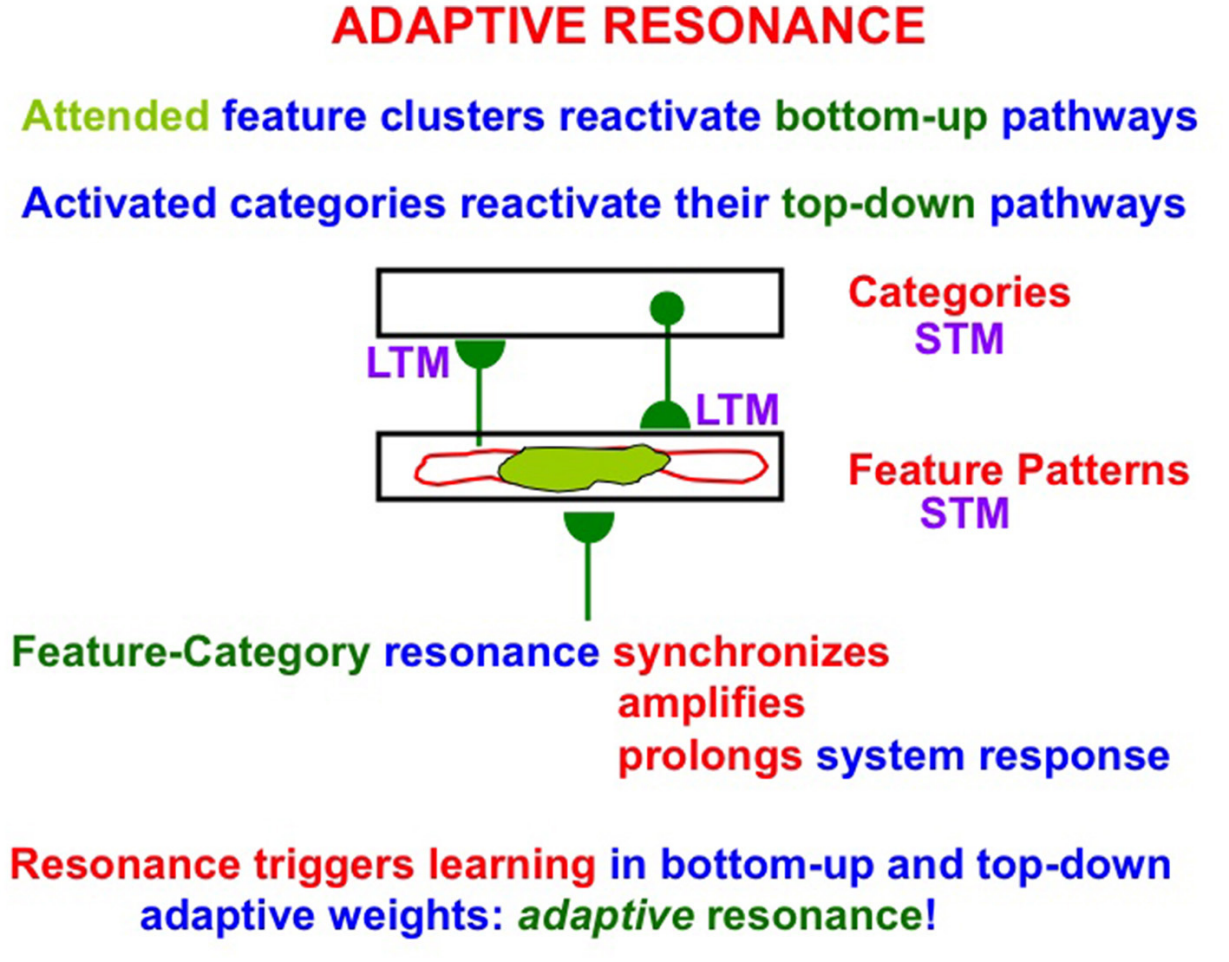
A two-level neural network of distributed feature patterns and learned categories as it experiences an adaptive resonance that is supported by signals in bottom-up adaptive filters and top-down learned expectations. The attended features (in light green) are the critical feature pattern. STM = cell activations or short-term memory (STM) traces. LTM = adaptive weights or long-term memory (LTM) traces. A *feature-category resonance* occurs when a good enough match exists between an active feature pattern and its recognition category. Such a resonance triggers fast learning in the LTM traces as well as conscious recognition of the object coded by the active feature pattern.

ART is trustworthy (because it is *explainable*) and *reliable* (because it *self-stabilizes learned memories*). To achieve these properties, ART incrementally learns via its adaptive weights, or long-term memory (LTM) traces, in its bottom-up adaptive filters and top-down expectations (Figure 1). Expectations are matched against input feature patterns. Expectations focus attention upon the patterns of *critical features* that are causal and control predictive success. A good enough match between bottom-up feature patterns and top-down expectations triggers an *adaptive resonance* that incorporates new information into previously existing recognition categories, or creates new recognition categories if inputs are too novel to be represented by established categories.

Apart from its explanatory success, why should a doubtful reader believe that ART is special? One reason is that I derived ART from a *thought experiment* in an oft-cited 1980 *Psychological Review* article (Grossberg, 1980). A thought experiment is the gold standard in providing a conceptually secure foundation for a scientific theory. Perhaps the most famous thought experiments in science were the ones that Albert Einstein used to derive both Special Relativity Theory and General Relativity Theory. My thought experiment asks how *any* system can *autonomously* learn to correct predictive errors in a changing world.

This thought experiment derives ART as the unique class of models that can do this from a few familiar facts of life that do not mention the mind or brain. ART is thus a *universal* solution to the problem of autonomous error correction in a changing world.

ART has been successfully used in large-scale applications in engineering, technology, and AI, where it beat other models in benchmark studies. Fielded applications include engineering design retrieval systems that include millions of parts defined by high-dimensional feature vectors, and that were used to design the Boeing 777 (Caudell et al., 1990, 1991, 1994; Escobedo et al., 1993). This Boeing team created the first dedicated ART optoelectronic hardware implementation (Caudell, 1992; Wunsch et al., 1993). Other applications include classification and prediction of sonar and radar signals, of medical, satellite, face imagery, and social media data, and of musical scores; control of mobile robots and nuclear power plants, air quality monitoring, remote sensing mapping, medical database prediction, strength prediction for concrete mixes, signature verification, tool failure monitoring, chemical analysis from ultraviolent and infrared spectra, frequency-selective surface design for electromagnetic system devices, and power transmission line fault diagnosis [see Grossberg (2021b), http://techlab.bu.edu/resources/articles/C5, and Da Silva et al. (2019) and Da Silva et al. (2020)].

These models, on which ChatSOME is built, exemplify *neural-symbolic computing*. As noted by Wang et al. (2024) “Neural-symbolic computing (NeSy), which pursues the integration of the symbolic and statistical paradigms of cognition, has been an active research area of Artificial Intelligence (AI) for many years. As NeSy shows promise of reconciling the advantages of reasoning and interpretability of symbolic representation and robust learning in neural networks, it may serve as a catalyst for the next generation of AI.” ChatSOME embodies neural-symbolic computing by synthesizing ART and related models (Carpenter and Grossberg, 1994; Grossberg, 1976b, 1980, 1987, 1988). Indeed, even within the simplest category-learning versions of ART, each learned category is a symbol, and all the learned categories, taken together, provide a representation of the recent statistics of the model's object and event learning in a changing world.

Colelough and Regli (2025) also provide a review of neuro-symbolic AI. They noted in their Abstract that “there is a notable gap in research focused on explainability and trustworthiness, which is critical for the deployment of reliable AI systems.” As I noted above, ART has been explainable and trustworthy since I introduced it in 1976. Those interested in the even earlier neural network history might like to know that I introduced the biological neural network paradigm in 1957 when I was a Freshman at Dartmouth College taking introductory psychology, and my short-term memory (STM), medium-term memory (MTM), and long-term memory (LTM) laws are still used to explain data about how brains make minds. That is why colleagues call me the Father of AI (see https://en.wikipedia.org/wiki/Stephen_Grossberg).

Another example of neural-symbolic computing, and one that uses a form of attention, is the Transformer model (Vaswani et al., 2017). Transformers are feedforward, and their attention mechanism differs from the ART Matching Rule, which characterizes attention in ART. The ART Matching Rule is embodied by a top-down, modulatory on-center, off-surround network, a prediction confirmed anatomically and neurophysiologically in multiple species (Grossberg, 2021a). Attention in ART stabilizes learned memories while focusing on learned critical feature patterns.

To the present time, six different adaptive resonances in different parts of our brains carrying out different psychological functions have been characterized and used to explain psychological and neurobiological data: *Surface-shroud resonances* support conscious seeing of visual objects and scenes; *Feature-category resonances* support conscious recognition of visual objects and scenes; *Stream-shroud resonances* support conscious hearing of auditory objects and scenes; *Spectral-pitch-and-timbre resonances* support conscious recognition of sources in auditory streams; *Item-list resonances* support conscious recognition of speech and language; and *cognitive-emotional resonances* support conscious feelings and recognition of their sources.

With these abilities in hand, an observer can learn to associate large numbers of learned language utterances with their scenic and emotional meanings with the help of a teacher, in the same way as in the study by Grossberg (2023). The main difference is that the observer has experienced *a huge repertoire of visual scenes to which language descriptors can be attached*. As a result, *the observer's brain can learn large language models and associate them with their many scenic and emotional meanings*.

We then need to explain.

### Storage and retrieval of large language models

How are large language models stored in an efficiently retrievable way in our brains? A learner's experiences in real time provide a scaffold for doing this. In particular, I will describe how individual language utterances can be sequentially organized in the order that a learner views different parts of a scene under a teacher's guidance.

This can be done in either of two ways:

First, a visual representation of each view can be associated with a sentence that describes this view, as in the study by Grossberg (2023).

Second, each sentence that is generated as a sequence of views is perceived and can be temporarily stored in *cognitive working memory* within the prefrontal cortex. The distributed representation of the sentence can then be compressed, or chunked, by learning at the next processing level into a recognition category, or *list chunk*, that responds selectively to the stored sentence with which it is associated. Multiple, sequentially activated list chunks can, in turn, be sequentially stored in a working memory, then chunked, at higher cortical levels (Bradski et al., 1992, 1994; Grossberg, 1978a,b, 2017, 2018, 2022, 2023; Grossberg and Pearson, 2008; Kazerounian and Grossberg, 2014; Silver et al., 2011).

**Figure 2:**
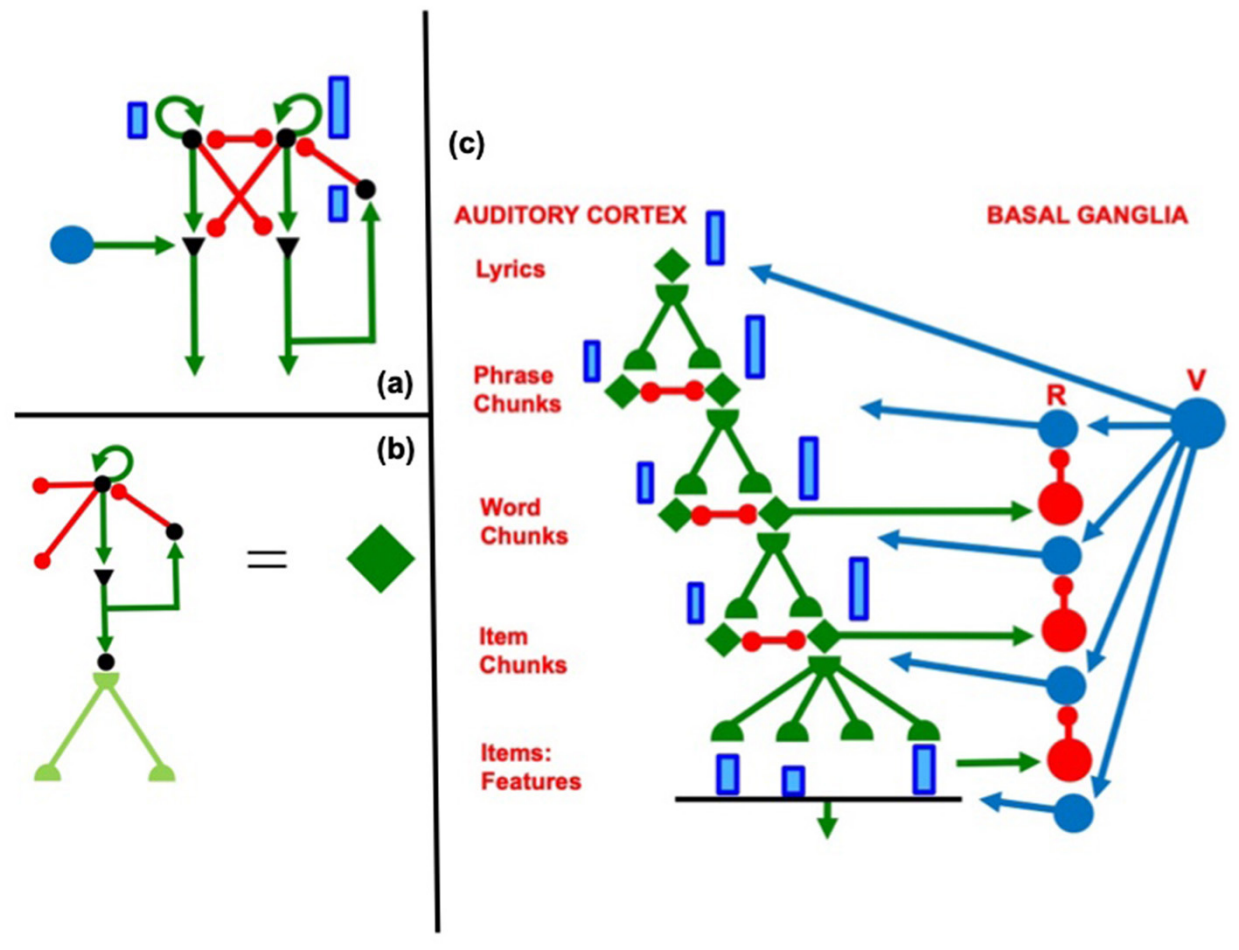
**(a)** When a rehearsal wave R (blue disk) turns on, the item that is stored in working memory can be rehearsed while it self-inhibits its working memory representation. Relative activity amplitudes are represented by the sizes of vertical blue rectangles. Triangular cells are polyvalent. **(b)** Filled diamond summarizes key stages in choosing items to be rehearsed and associating them with a bottom-up adaptive filter and learned top-down expectations. **(c)** Recursive read-out, under volitional control, from the hierarchy of processing stages that represents the lyrics of a song. Green represents excitatory connections. Red represents inhibitory connections. Blue disks represent volitional gain control signals. [Reprinted with permission from Grossberg (2022)].

In this hierarchical network, the nodes, or cell populations, in each working memory are list chunks of sequences that are stored in working memory at the previous level: the nodes at the second level of the hierarchy can represent list chunks of sentences at the first level, and the nodes at the third level of the hierarchy can represent ordered sentences as part of a story. Figure 2 shows how this can work during storage and learning of the lyrics of a song, starting at the level of acoustic features, as explained by Grossberg (2022).

Later in life, spontaneous visual exploration of a scene (Browning et al., 2009; Chang et al., 2014; Elder et al., 2009; Grossberg et al., 2012; Srihasam et al., 2009) enables previously, or newly, learned language descriptors of scenic views to be sequentially activated, stored, learned, and remembered as organized stories about the new scene, which can later be replayed at will, either subvocally in the learner's mind, or vocally to nearby listeners.

### Representing the places where, and the times when, events occur

The above processes help to explain how the place and the time that an event occurs are represented in the brain. Details about the place are *explicitly* included in these processes by recalling visual memories about the scene in which the event occurred. Details about time are *implicitly* included because, when a child asks you “When did mommy throw the ball?,” you can at least partially infer how long ago she threw the ball by recalling visual memories about how mommy was dressed and the objects in the scene and their arrangement. Visual representations of mommy moving in space occur within brain regions such as the visual, temporal, and prefrontal cortices. The next section, about contextually cued visual search, will review in greater detail how space and time are represented in this sense.

### Episodic learning and memory: neural relativity during entorhinal–hippocampal interactions

Episodic learning and memory provide another type of representation of the time when, and the place where, an event occurred (Baddeley, 2001; Baddeley et al., 2002; Burgess et al., 2002; Eichenbaum, 2017; Ezzyat and Davachi, 2011; Fletcher et al., 1997; Moscovitch et al., 2016; Schacter and Madore, 2016; Squire and Zola, 1998; Sugar and Moser, 2019; Tulving, 1983, 1993, 2002; Tulving and Thomson, 1973). As described by Google: “Episodic learning and memory involve encoding, storing, and retrieving specific, personal experiences, including details about time and place, and are distinct from semantic memory, which focuses on factual knowledge.”

Neurobiological experiments have suggested that interactions within and between the *entorhinal cortex* and the *hippocampal cortex*, among other brain regions, contribute to episodic learning and memory. I and several PhD students and postdocs have developed neural network models of the brain mechanisms within the *lateral* entorhinal–hippocampal system that learn *adaptively time* actions triggered by currently valued objects in a scene. We have also modeled the brain mechanisms within the *dorsal* entorhinal–hippocampal system that learn to *navigate the space* in which these valued objects occur.

**Figure 3:**
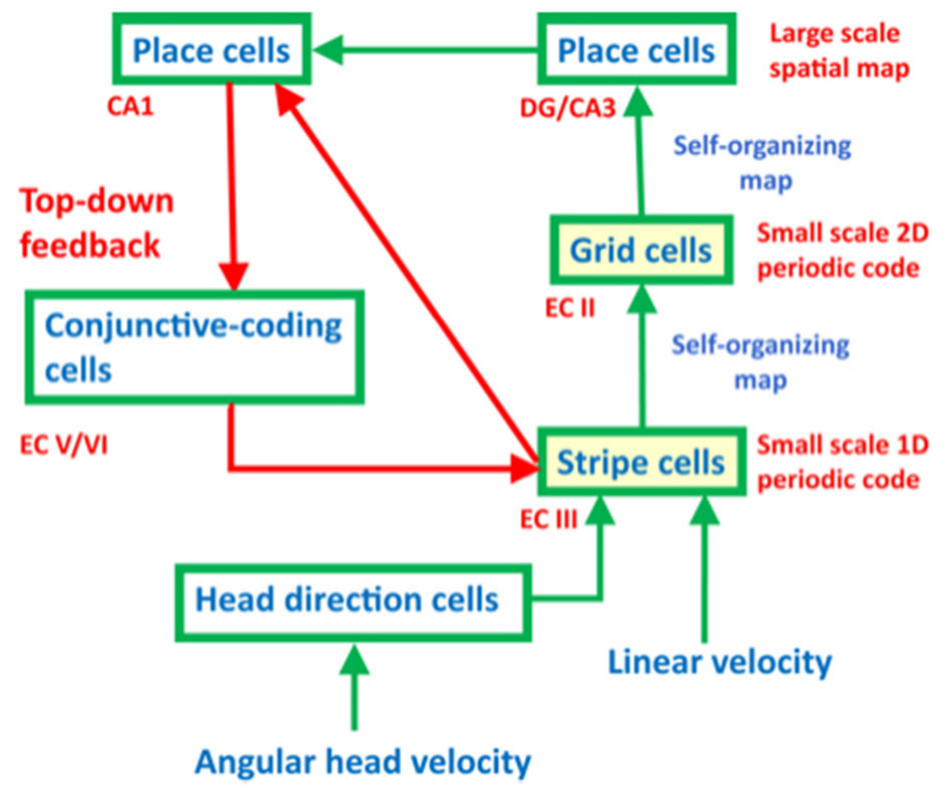
The entorhinal–hippocampal system has properties of an ART spatial category learning system, with hippocampal place cells as the spatial categories. [Reprinted with permission from Grossberg (2021b)].

One might immediately wonder: how did evolution discover computational machinery for representing space and time in this way? The GridPlaceMap neural model of entorhinal–hippocampal interactions proposes how these dorsal and ventral representations of space and time emerge from variations of the *same* circuit mechanisms. I like to call this exciting homology *neural relativity* to emphasize its unification of concepts about space and time (Gorchetchnikov and Grossberg, 2007; Grossberg, 2021b; Grossberg and Pilly, 2012, 2014). Remarkably, the GridPlaceMap model also proposes how both entorhinal grid cells and hippocampal place cells are learned during development as spatial categories in a hierarchy of self-organizing maps (SOMs), where SOMs are a basic building block of many kinds of brain processes, including perceptual, cognitive, and emotional processes (Figure 3). Moreover, grid cells and place cells can use the *same SOM equations* to learn their strikingly different receptive fields, the difference being due entirely to the different positions of these cells in the entorhinal–hippocampal hierarchy. In addition, both grid and place cells develop by detecting, learning, and remembering the most frequent and energetic co-occurrences of their different input patterns. Historical and comparative reviews of several influential cognitive and neural network models for learning, categorization, and decision-making, including SOMs, are provided in Grossberg (1984c, 1986, 1987, 1988). The top-down feedback pathway in Figure 3 dynamically stabilizes grid cell and place cell learning. The entire neural circuit is an ART system for learning spatial categories for navigation. Primordial ART mechanisms operate even during non-neural processes such as gastrulation in sea urchins, and illustrate a universal developmental code shared by all cellular organisms (Grossberg, 1978c, 2021b).

Once learned, these temporal and spatial representations within the ventral and dorsal entorhinal–hippocampal streams can be associated with representations of concurrently occurring visual scenes to form episodic memories, even while sentences that describe these scenes are associated with them. I will not use episodic memories in the remainder of this article. Two articles that use ART to model episodic memory in applications are Hu et al. (2022) and Wang et al. (2012).

I can now review the main additional process that is used in the extended ChatSOME model, followed by a brief review of several of the processes used in the original ChatSOME model. The reader who wants a more complete explanation of all the processes used in the original ChatSOME model can find it in the study by Grossberg (2023).

This additional ChatSOME process concerns how humans can learn to consciously perceive, attend, search, and understand large numbers of natural and man-made visual scenes throughout life. Language utterances can then be associated through learning with attended views of these scenes. Because the scenes include all our visual experiences, they provide a substrate for learning large language models and their perceptual and affective meanings.

## Learning to understand a scene during contextually cued visual search

### Eye movements and visual search: from gist to scene understanding

Humans make thousands of eye movements every day. Some eye movements explore scenes without any goals in mind. Other eye movements search for valued persons or objects that are expected to be found in a scene, such as finding a friend with whom you are having lunch in a restaurant, or locating your reserved seat in an auditorium before a play or concert starts. To search efficiently, our visual attention uses knowledge of what to expect and where to look for it (Neider and Zelinsky, 2006). Such knowledge comes either from external, or exogenous, cues, such as visual or verbal information about a target, or from internal, or endogenous, memories of spatial or object placements in a scene (Chun, 2000).

The *gist*, or first glance, of a scene provides a rapid, but coarse, initial representation of a scene, such as whether the scene is of a mountain range, beach, or city street. Gist helps human observers realize what kind of scene is being viewed before searching it with eye movements. Gist illustrates the fact that human observers process visual information in a global-to-local and coarse-to-fine manner (Navon, 1977; Schyns and Oliva, 1994). After the first glance of a novel image in ~200–300 ms, people can recognize the basic-level scene identity (Potter, 1975; Tversky and Hemenway, 1983) and surface properties (Oliva and Schyns, 2000; Rousselet et al., 2005), spatial structures (Biederman et al., 1974; Sanocki, 2003), and meanings (Potter, 1975; Potter et al., 2004) without yet attending individual objects in the scene. The gist of a scene hereby provides contextual guidance for where in the scene a search target may be located (Torralba et al., 2006).

The first-order approximation to scene understanding that gist provides is often followed by evidence accumulation about the scene to achieve a more detailed perceptual and cognitive understanding (Gold and Shadlen, 2007; Grossberg and Pilly, 2008; Heekeren et al., 2008; Irwin, 1991; Jonides et al., 1982). Neural models that I have developed with several collaborators clarify how successive spatial attention shifts and eye movements enable us to learn progressively more detailed understanding of scenes (Grossberg and Huang, 2009; Huang and Grossberg, 2010) and the objects within them (Fazl et al., 2009; Foley et al., 2012; Grossberg and Williamson, 1999). In particular, the neural model of Grossberg and Pilly (2008) provides a more powerful explanatory framework for perceptual decision-making than models based on Bayesian Inference (e.g., Gold and Shadlen, 2001, 2007; Knill and Pouget, 2004; Pouget et al., 2003), while also overcoming conceptual and explanatory weaknesses of the Bayes approach.

Since visual attention can be guided by cognitive and emotional control to objects or regions of interest, over and beyond scenic statistics or contexts, gaze locations and eye scanning paths also reflect task-dependent goals and internal drives (Ballard and Hayhoe, 2009; Hayhoe and Ballard, 2005; Rothkopf et al., 2007). For example, when geologists first walk into a desert, their attention may be attracted to the mineral deposits that they made the trip to analyze. However, if they are very thirsty when they arrive, their attention and actions may shift toward palm trees in an oasis where they can sate their thirst. Yarbus (1967) has provided a classic example of such goal-dependent scene search by recording eye movements for the same picture under different task instructions.

Due to how sequences of visual attention shifts are shaped by contextual constraints when viewing a scene, memories of the scene are not like a photograph, but rather emphasize attentionally salient scenic textures or objects (Kensinger et al., 2007). Bottom-up perceptual factors, top-down cognitive factors (Chen and Zelinsky, 2006; Leber and Egeth, 2006), and emotional factors (Armony and Dolan, 2002; Öhman et al., 2001) conjointly influence scene understanding.

A comprehensive neural model of how our brains achieve visual scene understanding must thus explain how exogenous and endogenous attention combine to organize scene perception and memory, and how evidence accumulation incrementally deepens awareness and knowledge of a scene during spatial attention shifts and scanning eye movements.

### ARTSCENE model

The ARTSCENE model (Grossberg and Huang, 2009) explains and simulates how spatial attention can regulate category learning and recognition of scenic textures, starting with global textures such as the gist of a scene, and then including increasing small spatial scales to refine identification of smaller textured regions over time.

**Figure 4:**
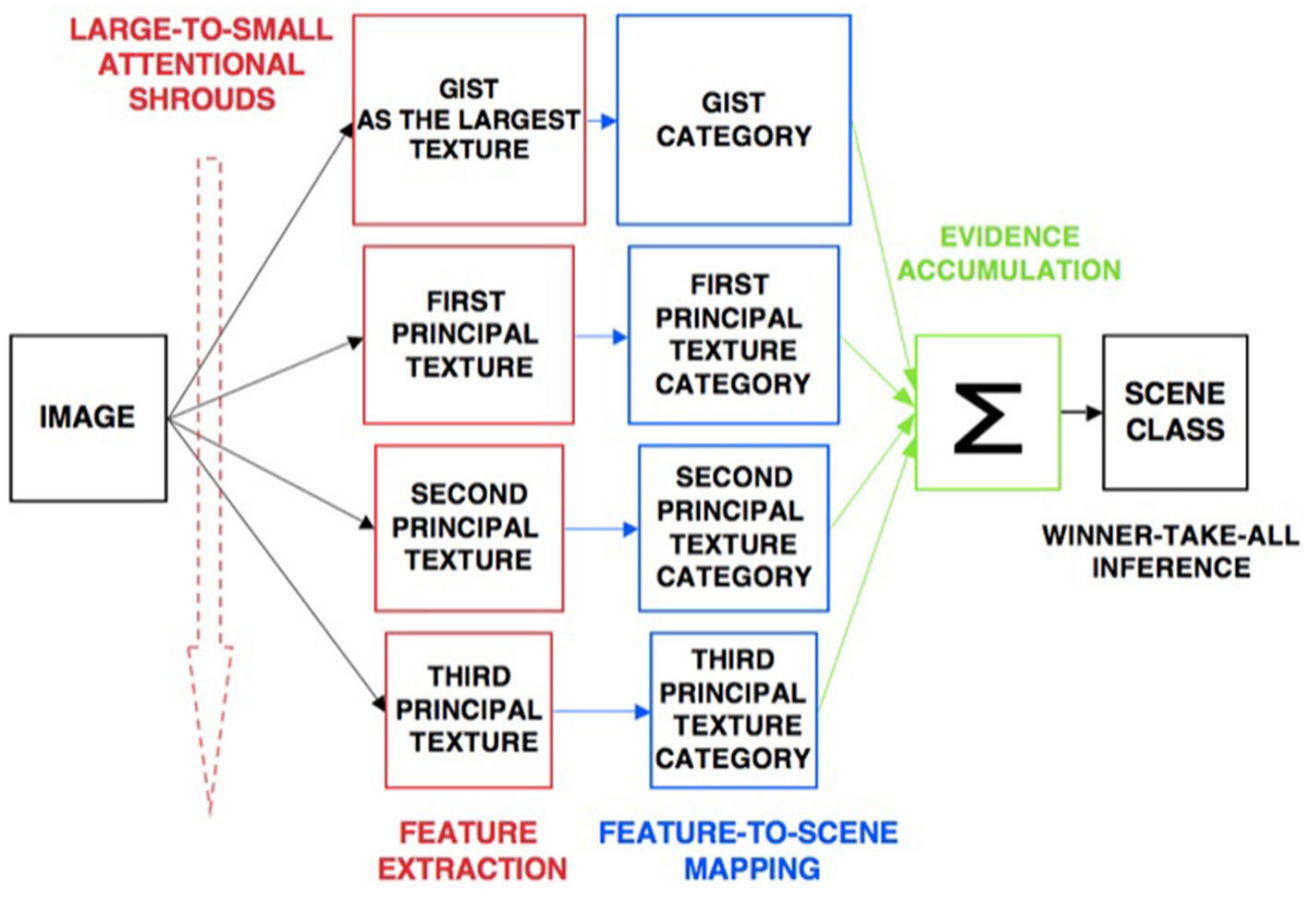
Classification of scenic properties as texture categories by the ARTSCENE model [Reprinted with permission from Grossberg (2021b)].

Scenic categories were learned in ARTSCENE using the ARTMAP model, which is capable of both unsupervised and supervised attention, fast learning without catastrophic forgetting, categorization, and prediction of non-stationary data and environments (Carpenter et al., 1991, 1992). After learning to categorize a scene's gist, scene identity was refined by assuming that the eyes randomly scan the scene, thereby landing in the largest textured region with the highest probability. An attentional spotlight at the position where the eyes land triggers a *surface-shroud resonance* that spreads spatial attention and conscious visual awareness across the attended region (Fazl et al., 2009), thereby enabling a texture category of that region to be learned. Then, the process is repeated, enabling ever-finer texture categories to be learned, until all the regions are classified (Figure 4).

**Figure 5:**
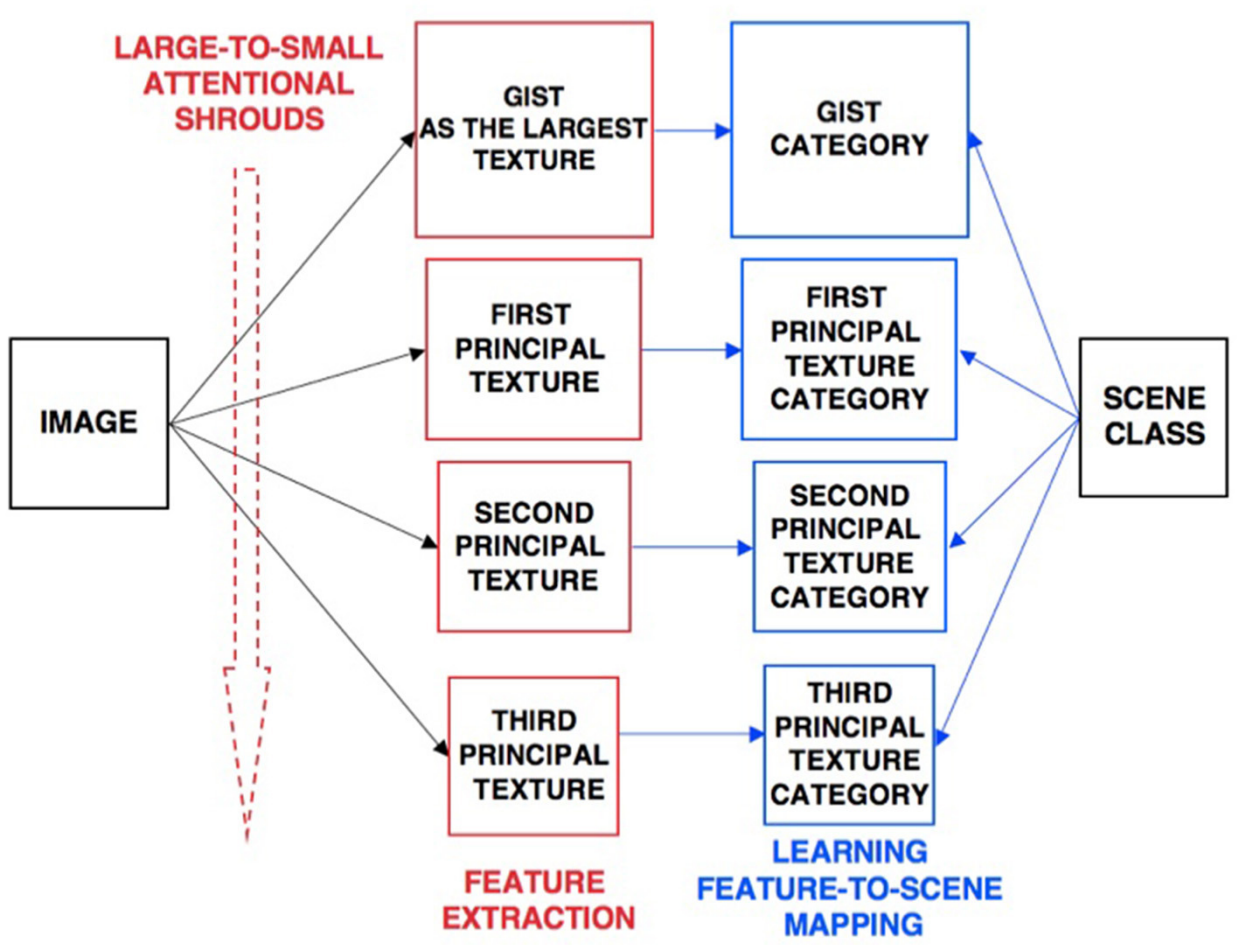
Voting in the ARTSCENE model achieves even better predictions of scene type. [Reprinted with permission from Grossberg (2021b)].

After learning was complete, recognition performance was determined by letting all the learned texture categories *vote* for the best scenic label (Figure 5). At the time ARTSCENE was published, it reduced the error rate of alternative scene classification models by 16.15%, even though these models were based on more complex and biologically implausible processes.

### ARTSCENE Search models scene understanding: object and spatial contexts influence search

After the ARTSCENE model was complete, Tren Huang and I frontally attacked the scene understanding problem by modeling the following more challenging competence: how scenic objects and their positions are learned and used to guide an efficient context-sensitive search for other objects in familiar types of scenes to learn the scene incrementally. For example, humans can learn that a certain combination of objects, such as a refrigerator and a stove, may define a context for a kitchen and use that knowledge to trigger an efficient search for another typical kitchen object, such as a sink, until the entire kitchen scene is learned.

The ARTSCENE Search model (Huang and Grossberg, 2010) was developed to understand the neural mechanisms of such memory-based context learning and guidance, and to explain challenging behavioral data. As in the ARTSCENE model, the ARTSCENE Search model simulates how a first glance of a scene learns a gist category. In addition, ARTSCENE Search triggers learning about both the object's identity and its position, while also matching learned top-down expectations against the object and its position to determine whether it is a target (e.g., a sink) or a non-target (e.g., a wall).

**Figure 6:**
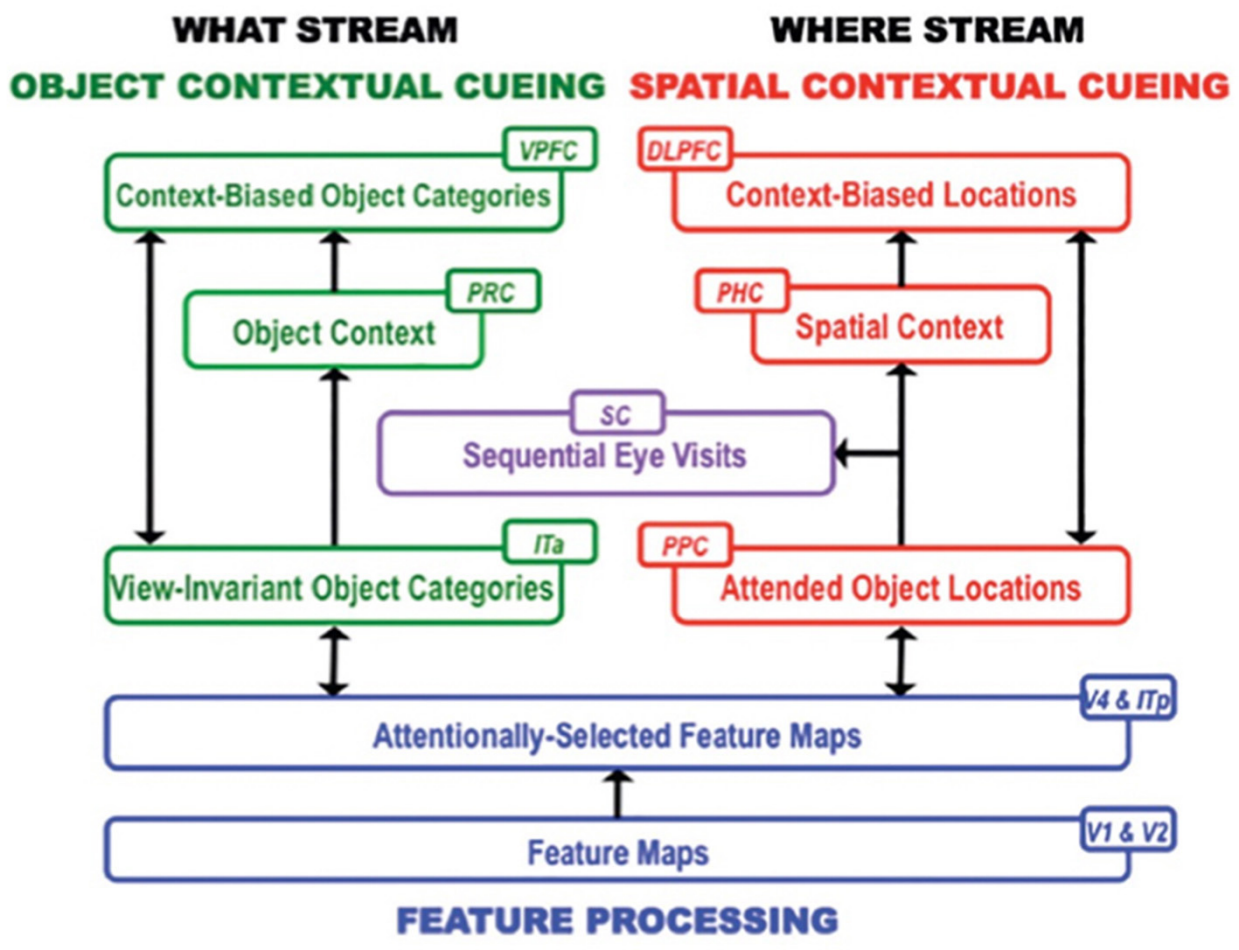
Macrocircuit of the ARTSCENE Search neural model for learning to search for desired objects by using the sequences of already experienced objects and their locations to predict what and where the desired object is. V1, First visual area or primary visual cortex; V2, Second visual area; V4, Fourth visual area; PPC, Posterior parietal cortex; ITp, Posterior inferotemporal cortex; ITa, Anterior inferotemporal cortex; MTL, Medial temporal lobe; PHC, Parahippocampal cortex; PRC, Perirhinal cortex; PFC, Prefrontal cortex; DLPFC, Dorsolateral PFC; VPFC, Ventral PFC; SC, Superior colliculus. [Reprinted with permission from Grossberg (2021b)].

This hypothesis is then incrementally refined as a scene is scanned with saccadic eye movements. Each eye movement adds to the *accumulated learned contextual evidence about object and spatial sequential contexts* that help to determine where to look next to most efficiently find the target. Sequences of the scene's object and positional representations are learned in this way through time. The model hereby simulates the interactive dynamics of object and spatial contextual cueing and attention in the cortical What and Where streams, starting from early visual areas through the medial temporal lobe to prefrontal cortex (Figure 6).

### Perirhinal and parahippocampal cortices store object and spatial contexts

Multiple brain regions cooperate to carry out these contextual processes (Figure 6). Sequences of fixated objects and their spatial positions are stored in object and spatial working memories within the model ventrolateral prefrontal cortex (VLPFC) and dorsolateral hippocampal cortex (DLPFC), respectively. Sequences of fixated objects and their positions are also stored in the model perirhinal cortex (PRC) and parahippocampal cortex (PHC), respectively. Stored PRC and PHC sequences define object and spatial *contexts* that interact with the VLPFC and DLPFC *working memories* via bottom-up adaptive filters. The proposed role of PRC and related cortical areas in defining *object* contexts, and of PHC and related cortical areas in defining *spatial* contexts, is supported by neuroimaging data in humans (Aminoff et al., 2007; Diana et al., 2007; Libby et al., 2014).

**Figure 7:**
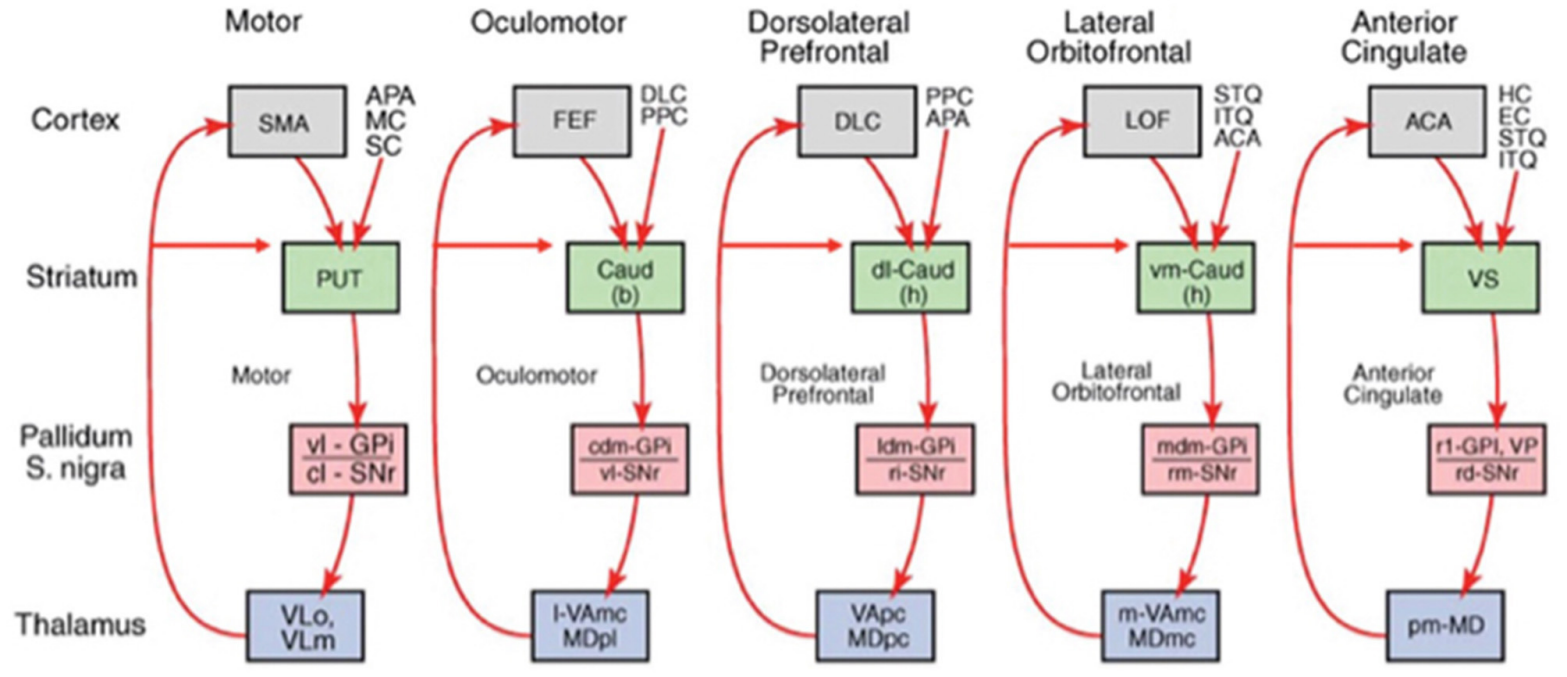
The basal ganglia gate neural processing in many parts of the brain. The feedback loop through the lateral orbitofrontal cortex (blue arrow) is the one that MOTIVATOR models (see Figure 9 for what MOTIVATOR is). [Reprinted with permission from Grossberg (2021b)].

Associative learning occurs in the ARTSCENE Search model, from a stored object or position in PRC or PHC to a stored object or position in VLPFC or DLPFC, respectively. This learning is modulated by a dopamine burst from the model basal ganglia (Figure 7) when a target is foveated and reinforced. In this way, predictively successful associations between PRC and VLPFC, and between PHC and DLPFC, can amplify the stored working memory items and list chunks that led to predictive success. The spatial attentional focus can be broadened or narrowed in a task-specific way to determine what objects or positions will influence the winning prediction.

By modeling these processes, ARTSCENE Search quantitatively simulated psychophysical data from experiments in the literature on contextual cueing, including spatial and object cueing, positive and negative spatial cueing, and local and distant cueing effects (e.g., Brockmole et al., 2006; Brockmole and Henderson, 2006; Chun, 2000; Chun and Jiang, 1998; Jiang and Wagner, 2004; Lleras and von Mühlenen, 2004; Olson and Chun, 2002).

After scene learning, the model's dorsolateral prefrontal cortex (area 46) primes possible object *positions* in the posterior parietal cortex based on goal-modulated percepts of spatial context that are represented in parahippocampal cortex. At the same time, the ventral prefrontal cortex (area 47/12) primes possible object *identities* in inferior temporal cortex based on the history of viewed objects represented in perirhinal cortex. Remarkably, the parahippocampal cortex and perirhinal cortex play computationally *complementary* roles (Grossberg, 2000) in spatial and object contextual processing. Grossberg (2021b) provides a more detailed summary of this scene learning process, including the functional roles of all the anatomical regions depicted in Figure 6.

### Organizing learned language utterances about a scene into a story

Once scene understanding is available, sentences that describe specific visual views of a scene can be associated with them using bidirectional associative learning; that is, learning from the sentence to the scene, as well as learning from the scene to the sentence. Sequential visual recall of scenic views can then enable the sentences to be recalled in the order in which the scene was scanned. As this happens, the sentences can be organized into stories using the multi-level network depicted in Figure 1. Either visual exploration or imagined visual recall of the scene can activate and recall brain representations of the associated sentences in the correct order. Alternatively, the learned linguistic story can be recalled from memory and thereby activate recall of the sequences of scenic views that the story describes.

This completes my heuristic summary of how a large language model can be learned and associatively linked to its perceptual meanings. How feelings are aroused uses the same brain processes that I described in the study by Grossberg (2023).

For completeness, I include an overview in the following sections of the review of the additional brain processes that were described in greater detail in Grossberg (2023) and that are needed to learn language utterances and associate them with the percepts and feelings that they describe.

## Several learning processes link language to perception and emotion

### Visual and auditory circular reactions enable a child to look where mommy is looking

How does a baby know where to look? Before a child can learn from an adult, the child must be able to pay attention to and learn to recognize the adult's face from multiple viewpoints when he or she speaks.

One early process that is needed to do this is a *visual circular reaction*. During a visual circular reaction, babies endogenously babble, or spontaneously generate, hand/arm movements to multiple positions around their bodies. Babbled movements endogenously sample the workspace within which a baby can reach. As their hands move in front of them, their eyes reactively look at their hands. While the baby's eyes look at its hands, an associative map is learned from its hand positions to the corresponding eye positions, and from its eye positions to hand positions. The learned maps between eye and hand in both directions are the “circular” reaction. After map learning occurs, when a person looks at a target position with their eyes, this eye position can use the learned associative map to prime the activation of a movement command to reach the target position in space. A volitional GO signal from the basal ganglia activates the reach.

An *auditory circular reaction* occurs during its own babbling phase. During an auditory circular reaction, babies endogenously babble simple sounds that sample the workspace of sounds that they can create. The babies also hear the sounds that they create. When the motor commands that caused the sounds and the auditory representations of the heard sounds are simultaneously active in the baby's brain, a map is learned between these auditory representations and the motor commands that produced them. After enough map learning occurs, a child can use the map to approximately imitate sounds from adult speakers. It can then incrementally learn how to speak using increasingly complicated speech and language utterances, again under volitional control.

These processes enable babies to learn to imitate simple sentences that adult caregivers say, such as “Mommy walk,” “Mommy throw ball,” and so on.

Learned capabilities, such as being able to look at objects in space, talk about them, and act upon them, provide a scaffold for learning about these objects. Grossberg (2021b, 2023) describe these processes in detail.

Learning to recognize an object such as mommy's face from many viewpoints means that the child learns an *invariant recognition category* of her face. *View-specific categories* of her face must also be learned which are activated when specific views of her face are seen, as mommy looks at something. These invariant and view-specific representations reciprocally interact with each other via learned associations, so that the child can invariantly recognize mommy's face *and* a view of it that predicts where she is currently looking. Then, the child learns to look where mommy is looking.

**Figure 8:**
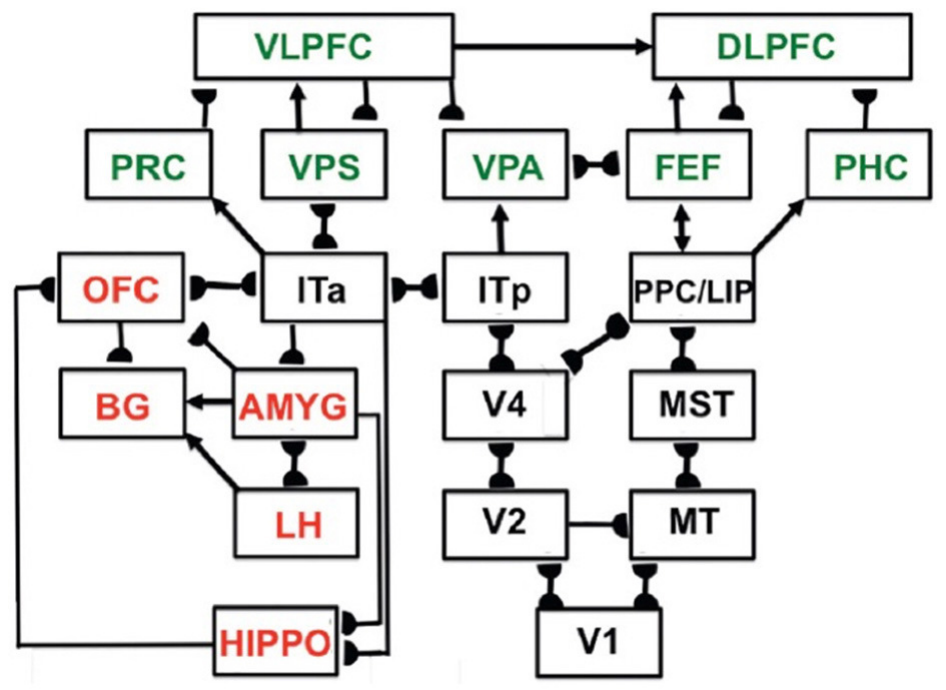
Macrocircuit of the main brain regions, and connections between them, that are modeled in the unified *predictive adaptive resonance theory* (pART) of cognitive-emotional and working memory dynamics. Abbreviations in red denote brain regions used in cognitive-emotional dynamics. Abbreviations in green denote brain regions used in working memory dynamics. Black abbreviations denote brain regions that carry out visual perception, learning and recognition of visual object categories, and motion perception, spatial representation, and target tracking. Arrows denote non-adaptive excitatory synapses. Hemidiscs denote adaptive excitatory synapses. Many adaptive synapses are bidirectional, thereby supporting synchronous resonant dynamics among multiple cortical regions. The output signals from the basal ganglia that regulate reinforcement learning and gating of multiple cortical areas are not shown. Moreover, not shown are output signals from cortical areas to motor responses. V1, striate, or primary, visual cortex; V2 and V4, areas of prestriate visual cortex; MT, middle temporal cortex; MST, medial superior temporal area; ITp, posterior inferotemporal cortex; ITa, anterior inferotemporal cortex; PPC, posterior parietal cortex; LIP, lateral intraparietal area; VPA, ventral prearcuate gyrus; FEF, frontal eye fields; PHC, parahippocampal cortex; DLPFC, dorsolateral hippocampal cortex; HIPPO, hippocampus; LH, lateral hypothalamus; BG, basal ganglia; AMGY, amygdala; OFC, orbitofrontal cortex; PRC, perirhinal cortex; VPS, ventral bank of the principal sulcus; VLPFC, ventrolateral prefrontal cortex. [Reprinted with permission from Grossberg (2021b)].

These invariant and view-specific categories are learned within the child's inferotemporal cortex, with view-specific learning in the posterior inferotemporal cortex (ITp) and invariant learning in the anterior inferotemporal cortex (ITa; Cao et al., 2011; Fazl et al., 2009). Figure 8 shows ITp and ITa within the more comprehensive neural architecture that I call predictive ART, or pART, which I describe below.

### Cognitive-emotional interactions focus motivated attention upon mommy's face

Why does the baby want to look at mommy's face at all? A baby typically learns its mommy's face while mommy carries out actions that reward the baby, such as feeding it with her breast or a bottle. The milk, comfort, warmth, etc., that are experienced during feeding are positively rewarding. As an invariant category of mommy's face is learned, it is bidirectionally associated with positive emotional centers, also called *drive representations* or *value categories*, that are activated in the baby's brain by mommy's rewarding activities. These drive representations are in the amygdala/hypothalamic system.

**Figure 9:**
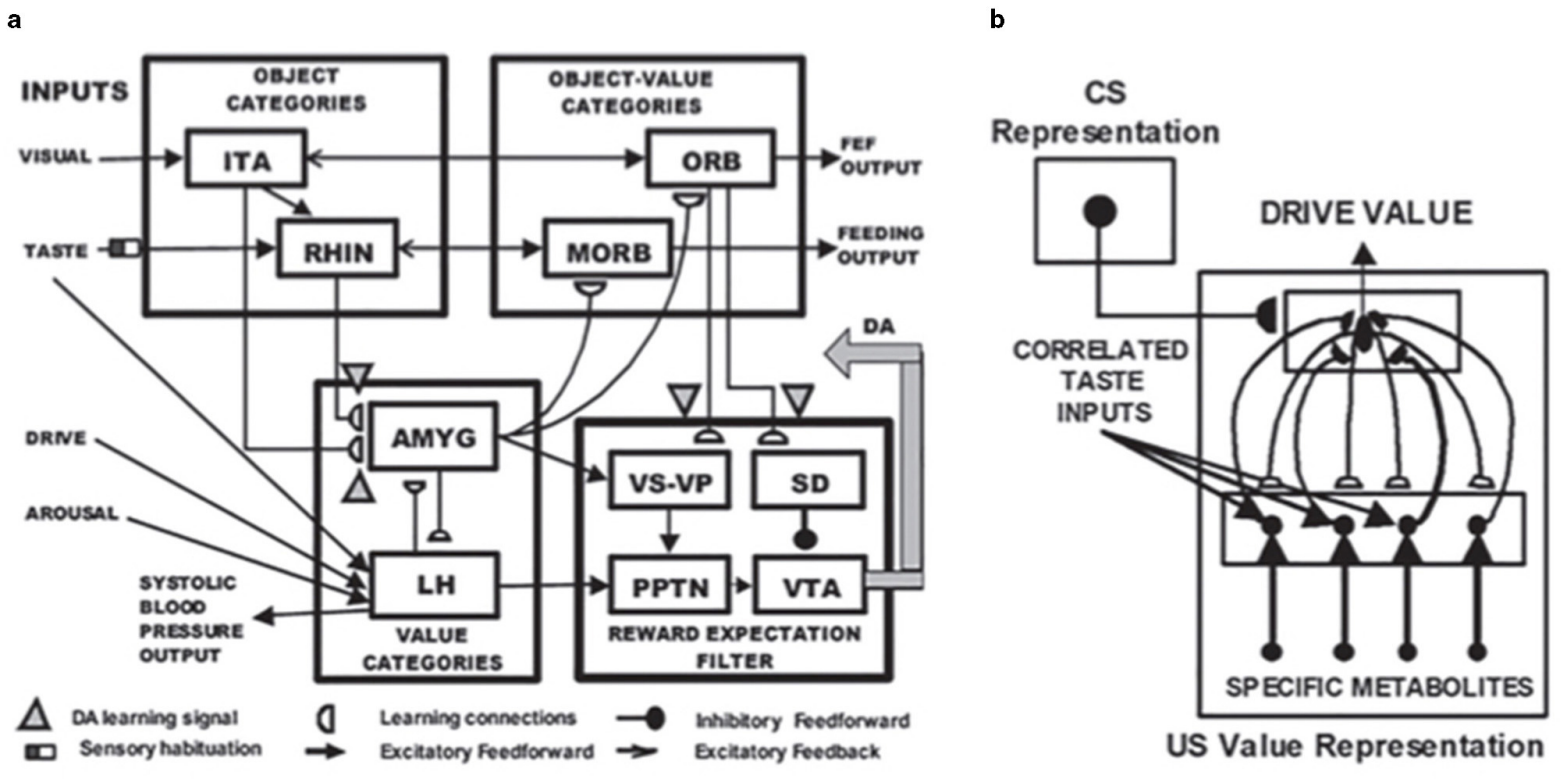
**(a)** The MOTIVATOR neural model generalizes CogEM by also including the basal ganglia. It can hereby explain and simulate complementary functions of the amygdala and basal ganglia (SNc) during conditioning and learned performance. The basal ganglia generate Now Print signals in response to *unexpected* rewards. These signals modulate the learning of new associations in many brain regions. The amygdala supports motivated attention to trigger actions that are *expected* to occur in response to conditioned or unconditioned stimuli. Object categories represent visual or gustatory inputs in the anterior inferotemporal (ITA) and the rhinal (RHIN) cortices, respectively. Value categories represent the value of anticipated outcomes on the basis of hunger and satiety inputs, in amygdala (AMYG) and lateral hypothalamus (LH). Object-Value Categories resolve the value of competing perceptual stimuli in medial (MORB) and lateral (ORB) orbitofrontal cortex. The Reward Expectation Filter detects the omission or delivery of rewards using a circuit that spans ventral striatum (VS), ventral pallidum (VP), striosomal delay (SD) cells in the ventral striatum, the pedunculopontine nucleus (PPTN), and midbrain dopaminergic neurons of the substantia nigra pars compacta/ventral tegmental area (SNc/VTA). The circuit that processes CS-related visual information (ITA, AMYG, and ORB) operates in parallel with a circuit that processes US-related visual and gustatory information (RHIN, AMYG, and MORB). **(b)** Reciprocal adaptive connections between the lateral hypothalamus and amygdala enable amygdala cells to become learned value categories. The bottom region represents hypothalamic cells, which receive converging taste and metabolite inputs, whereby they become taste-driven cells. Bottom-up signals from activity patterns across these cells activate competing value categories, or US Value Representations, in the amygdala. A winning value category learns to respond selectively to specific combinations of taste-drive activity patterns and sends adaptive top-down priming signals back to the taste-drive cells that activated it. CS-activated conditioned reinforcer signals are also associatively linked to value categories. Adaptive connections end in (approximate) hemidiscs. [Reprinted with permission from Grossberg (2021b)].

A neural model of *cognitive-emotional resonances*, called the Cognitive-Emotional-Motor, or CogEM, model, and its MOTIVATOR model generalization (Figure 9) that includes the basal ganglia, has been incrementally developed to achieve an ever broadening interdisciplinary explanatory range (e.g., Chang et al., 2014; Dranias et al., 2008; Fiala et al., 1996; Grossberg, 1971, 1972a,b, 1974, 1975, 1978d, 1982, 1984a,b, 2018, 2019a; Grossberg et al., 2008; Grossberg and Levine, 1987; Grossberg and Schmajuk, 1987, 1989). A cognitive-emotional resonance links attended valued objects to conscious feelings about them. In particular, the model includes a positive feedback loop that associates an invariant object category with an active drive representation. When it is activated for a sufficiently long duration, this positive feedback loop generates conscious feelings about the object while maintaining motivated attention on it and reading out commands for actions that can realize currently valued goals to acquire or otherwise manipulate the object.

The positive feedback during a cognitive-emotional resonance amplifies the activity of both the attended invariant object category and its view-specific categories. These amplified category representations can draw spatial attention to their position in space. As a result, when the baby and its mommy are in different spatial locations, the baby's attention can be drawn to attend to mommy's face. The interaction from an invariant category to its position in space has been modeled by the ARTSCAN Search model (Chang et al., 2014), among other properties.

The foundations of CogEM and MOTIVATOR for modeling our brain's cognitive-emotional dynamics, including reinforcement learning, were laid in Grossberg (1971, 1972a,b, 1974, 1975). My colleagues and I began to publish mathematical explanations and quantitative simulations of reinforcement learning data as soon as sufficiently powerful computers became available (e.g., Grossberg and Gutowski, 1987; Grossberg and Levine, 1987; Grossberg and Merrill, 1992, 1996; Grossberg and Schmajuk, 1987, 1989).

This explanatory range is not possible using the Temporal-Difference Learning model of Sutton and Barto (1987, 1998), which has been used primarily in applications.

### Comparing Nobel and Turing Prizes: Barto, Sutton, Hinton, Hopfield, Kahneman, and Tversky

The work of Sutton and Barto on reinforcement learning calls attention to Turing and Nobel Prizes for contributions that overlap with the models I developed, usually earlier than the award-winning work.

Andrew Barto and Richard Sutton won the Turing Award in 2025 for their work on reinforcement learning, which started in 1981. I listed above neural network models of reinforcement learning that I published between 1971 and 1975, despite the fact that the New York Times claimed on 5 March 2025 that “They are the undisputed pioneers of reinforcement learning” (https://lnkd.in/eiFc9HgD).

Geoffrey Hinton won the Turing Award in 2018 and the Nobel Prize in 2024 for his work on back propagation and deep learning. His 2024 Nobel Prize was for “foundational discoveries and inventions that enable machine learning with artificial neural networks.” Deep learning uses back propagation as its learning algorithm.

Back propagation was discovered by Amari (1972), Werbos (1974, 1994), and Parker (1982, 1985, 1986, 1987), reaching its modern form and being successfully simulated in applications by Werbos (1974). The algorithm was then popularized in 1986 in an article by David Rumelhart, Geoffrey Hinton, and Ronald Williams (Rumelhart et al., 1986).

As I noted above, in 1988 (Grossberg, 1988), I listed 17 fundamental computational problems that back propagation, and thus deep learning, have and that Adaptive Resonance Theory never had since its inception in 1976 [see also the review in the study by Grossberg (2020)]. These problems can be traced to the fact that back propagation is a feedforward adaptive filter, including that it is *untrustworthy* (because it is not *explainable*) and *unreliable* (because it can experience *catastrophic forgetting* at any stage of the hundreds or thousands of slow learning trials that are needed to complete its learning). It should thus never be used in life-or-death applications such as financial or medical applications. Back propagation also uses a non-biological, non-local weight transport to learn its adaptive weights, thereby excluding it as a plausible model of brain learning.

John Hopfield shared the 2024 Nobel Prize with Hinton. I published articles in 1967–1972 in the *Proceedings of the National Academy of Sciences*, which introduced the Additive Model that Hopfield (1984) used. My articles proved global theorems about the limits and oscillations of Generalized Additive Models, e.g., Grossberg (1967, 1968, 1971). These theorems provided a rigorous function for my research program to discover and develop biological neural networks that explain lots of psychological and neurobiological data. I proved more global theorems in the study by Grossberg (1978c,e). In the study by Grossberg (1978e), I also introduced a Lyapunov functional to help prove that sustained oscillations persist.

This mathematical foundation led Michael Cohen and me to discover in 1980, and finally manage to publish in 1982 and 1983 (e.g., Cohen and Grossberg, 1983), a Liapunov function that works for both the Additive Model and the Shunting Model. We used this Liapunov function to prove global convergence of our Liapunov function. Hopfield (1984) used a special case of our Liapunov function and did not prove global convergence. I was told that Hopfield knew our results before he published, but did not cite us [see Carpenter et al. (1987)].

Amos Tversky and Daniel Kahneman developed Prospect Theory to model how humans make irrational decisions when faced with risky probabilistic alternatives (Kahneman and Tversky, 1979), for which they won the Nobel Prize in 2002. Prospect Theory uses formal algebraic rules to fit human decision-making data. William Gutowski and I explained and simulated their data with Affective Balance Theory (Grossberg and Gutowski, 1987), which uses the brain's cognitive-emotional interactions that I published between 1971 and 1975. In this sense, my CogEM model predicted their data. We also explained data about preference reversals that Prospect Theory cannot.

Our model also answers the question: if evolution selects adaptive behaviors for survival during Natural Selection, then why are so many decisions irrational and even self-defeating? Our model shows how adaptive mechanisms for cognitive-emotional interactions can break down when risky probabilistic alternatives exist. They work most, but not all, of the time.

### Joint attention: how viewing a valued face triggers learned orienting to an attended object

**Figure 10:**
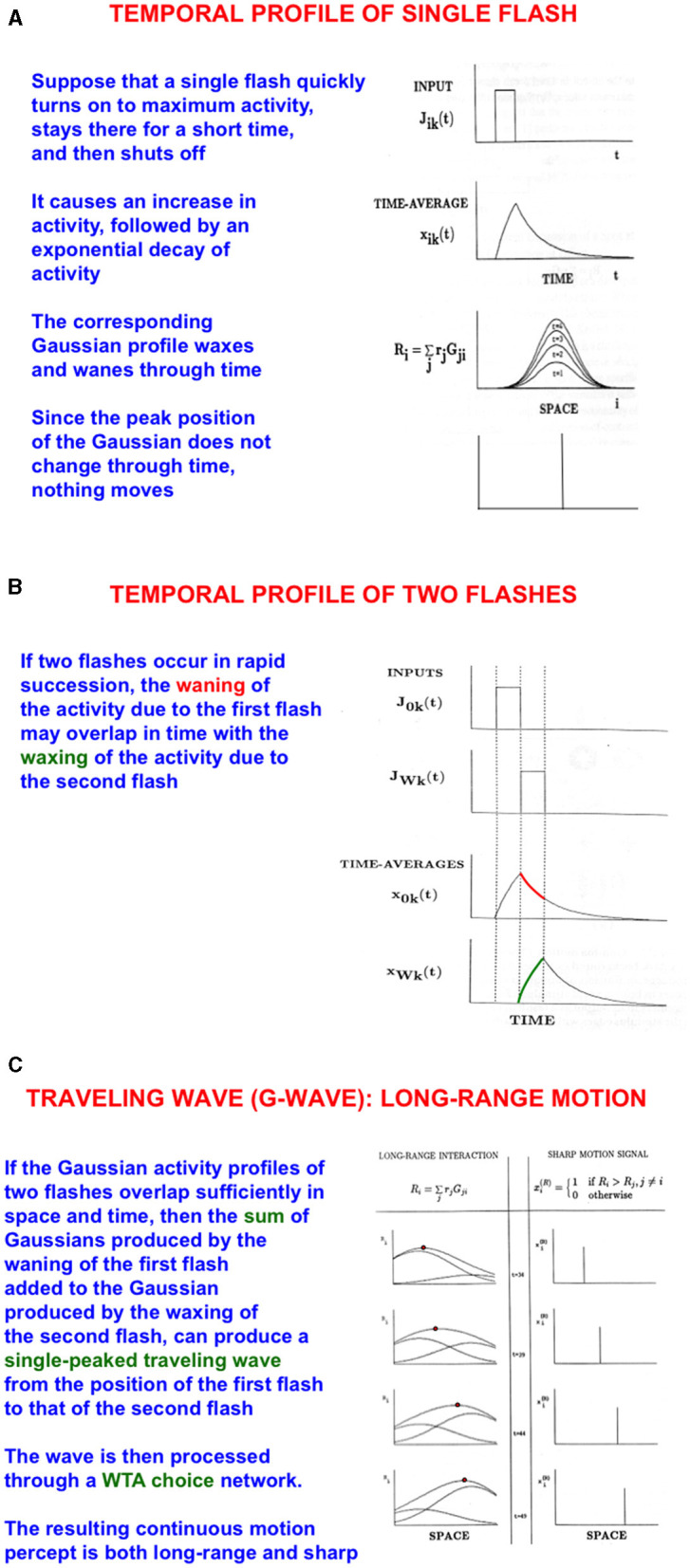
Long-range apparent motion: the sum of the waning Gaussian activity profile due to the first flash (at mommy's face) and the waxing Gaussian activity profile due to the second flash (at mommy's hand) has a maximum that moves like a traveling wave from the first to the second flash. In greater detail: **(A)** As a flash waxes and wanes through time, so too do the activities of the cells in its Gaussian receptive field. Because the maximum of each Gaussian occurs at the same position, nothing is perceived to move. **(B)** If two flashes occur in succession, then the cell activation that is caused by the first one can be waning while the activation due to the second one is waxing. **(C)** The sum of the waning Gaussian activity profile due to the first flash and the waxing Gaussian activity profile due to the second flash has a maximum that moves like a traveling wave from the first to the second flash. [Reprinted with permission from Grossberg (2021b)].

How does a baby learn to associate an attended view of mommy's face with the position in space where she is looking or pointing? As mommy points her arm and hand to an object for the baby to attend, spatial attention in the baby's brain can flow from mommy's attended face representation along her arm to her hand. Such a flow of spatial attention is an example of *long-range apparent motion*. I have called this flow a G-wave, or Gauss-wave (Figures 10A–C), because it describes how attention flows as a bump of Gaussianly shaped activity from its initial position to its final position (Francis and Grossberg, 1996; Grossberg, 2014; Grossberg and Rudd, 1989, 1992). In the current example, spatial attention flows from mommy's face to her moving hand.

### Learning to associate a view of mommy's face with the position of her hand in space

As noted above, a G-wave can travel from mommy's face to her hand as she points at an object of interest. An association can then be learned from the view-specific category of mommy's face to the attended final position of her hand in space. This view-specific category can then activate the learned association to predict where mommy is looking, so the baby can look in the direction that mommy is looking.

**Figure 11:**
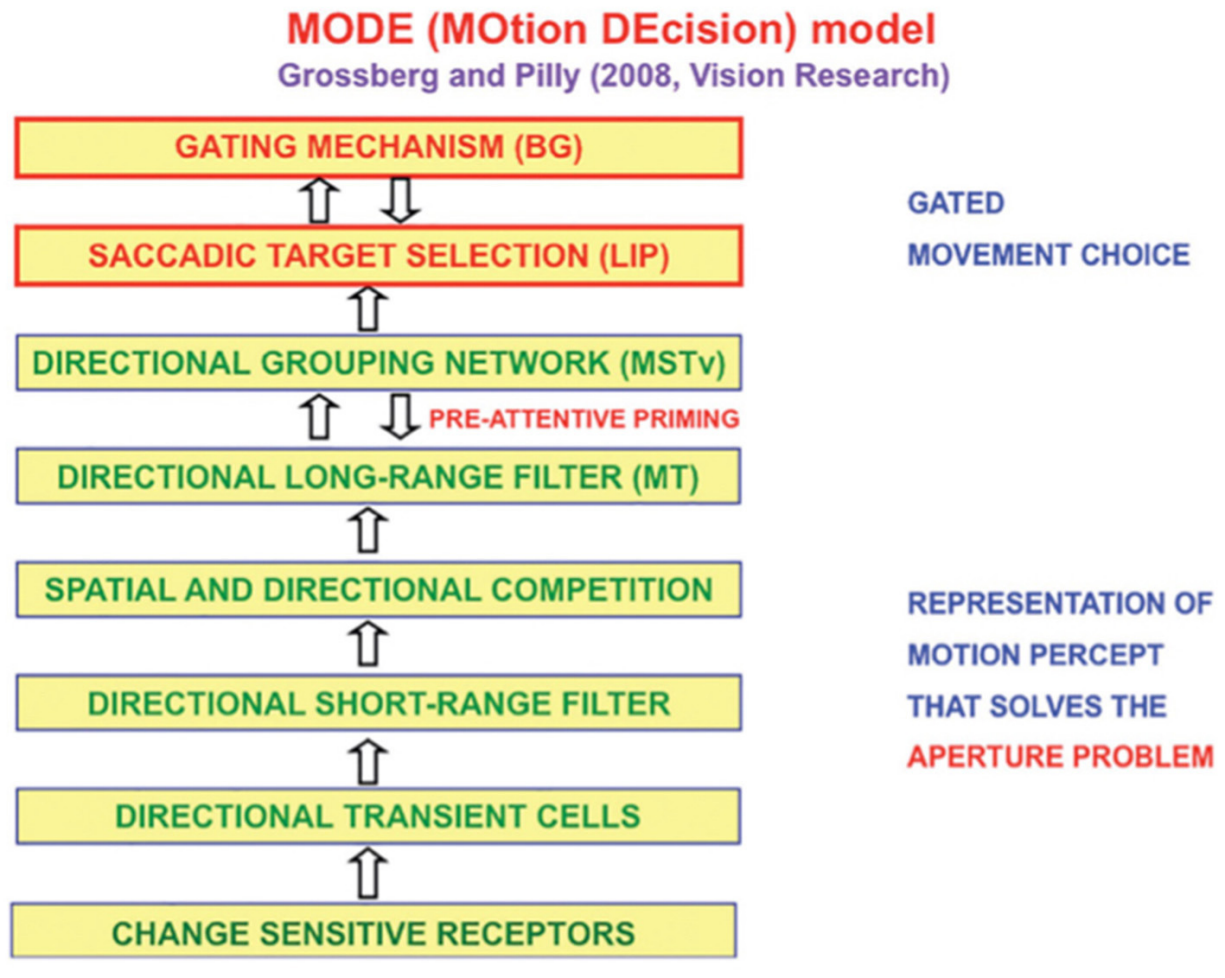
The MODE model uses motion preprocessing stages, collectively called the Motion BCS [green letters], as its front end, followed by a saccadic target selection circuit in the model LIP region [red letters] that converts motion directions into movement directions. These movement choices are also under basal ganglia (BG) control. MT, Middle Temporal area; MSTv, ventral Middle Superior Temporal area; LIP, Lateral Intra-Parietal area. [Reprinted with permission from Grossberg (2021b)].

The MOtion DEcision, or MODE, model of Grossberg and Pilly (2008) explains how the direction of mommy's motion is converted into saccadic eye movements that maintain fixation where mommy is looking (Figure 11).

### Learning to associate mommy's face with her name builds on an auditory circular reaction

As mommy's invariant face category is learned, the baby can also learn to associate it with an auditory production of mommy's name. This ability builds on the auditory circular reaction. If mommy responds positively to hearing her name, the child's resultant cognitive-emotional interactions strengthen the learned association between seeing mommy and saying mommy.

### Learning to recognize mommy's movements

Before a child can learn short sentences such as “mommy points” or “mommy walks,” the child must first learn to recognize her movements and learn names for them. Multiple perceptual processes in both the form and the motion cortical streams cooperate to enable this to happen. They are described in greater detail in Grossberg (2021b) and Grossberg (2023). Here, I briefly summarize why a lot of the visual cortex is needed to do this kind of computation.

A series of changing positions of a moving form, such as mommy, is computed in the What cortical stream. Perceiving a series of an object's changing positions is not, however, the same thing as perceiving its motion. Object motion is computed in the Where cortical stream. Form and motion are computed in separate cortical streams because object form is sensitive to the *orientation* of an object's boundaries, whereas object motion is sensitive to an object's *direction* of motion. Computation of motion direction pools directional estimates from all the different orientations of an object's boundaries that move in the same direction. A computation of motion direction hereby eliminates the information that computes object orientation. I have shown elsewhere that these parallel computations of object form and object motion are *computationally complementary* (Grossberg, 1991).

The Where stream needs a complete visual representation of an object's form to successfully track it. A representation of object form in the What stream is topographically mapped into a representation of its motion in the Where cortical stream, whose dynamics can track it through time. The 3D FORMOTION model simulates how this happens (Berzhanskaya et al., 2007).

Moreover, when a complex object such as mommy walks or points, different parts of her body move in different directions and speeds. Our brains compute the motion direction of mommy's body, and the motions of her legs and arms *relative to* her body, as she walks. This can be done using *vector decomposition* by a recurrent on-center off-surround network that occurs throughout our brains (Grossberg et al., 2011).

As mommy walks, her leg that is further from the child is partly occluded by the closer leg. A complete perception of the partially occluded leg is accomplished by the process of 3D figure-ground separation, whereby all objects in a scene are separated in depth. The 3D FORMOTION model explains how this happens (Berzhanskaya et al., 2007). Mommy's completed representations can then be recognized by the child's brain as it computes their motion directions and speeds.

### Nouns and verbs: learning to say “mommy walks left” while observing her move

How does a child's brain learn both a perceptual category and a language category for “walk” and “walking”? Where in the brain are the perceptual and linguistic representations of the verb “walk(s)” in “mommy walk(s)” represented?

First, consider the perceptual representation. Here, a view, or succession of views, of mommy standing up with her legs on the ground in a characteristic walking pose is classified. Large receptive fields average across these details to extract mommy's overall shape and silhouette.

Multiple oriented scales, or filter sizes, from fine through coarse, initially process all incoming visual information. Higher-order processing stages select the scales that are most informative in different situations by associating them with their predictive consequences. Only informative scales will learn strong associations. Finer scales will learn to categorize mommy's facial views, while coarser scales learn to categorize actions such as walking.

Suppose that the co-occurrence of two perceptual categories—of mommy's face and her walk pose—together triggers learning of a category that selectively fires when mommy walks. This conjunctive category can be associated via learning with the heard utterance “mommy walks” or “mommy is walking” via a bi-directional associative map.

A single pose of walking is often enough to recognize walking, just as a single pose of standing is enough to recognize that posture. Recognition of movements that cannot be effectively categorized using a single pose requires the Where cortical stream.

### Learning to recognize and track mommy's movement direction

**Figure 12:**
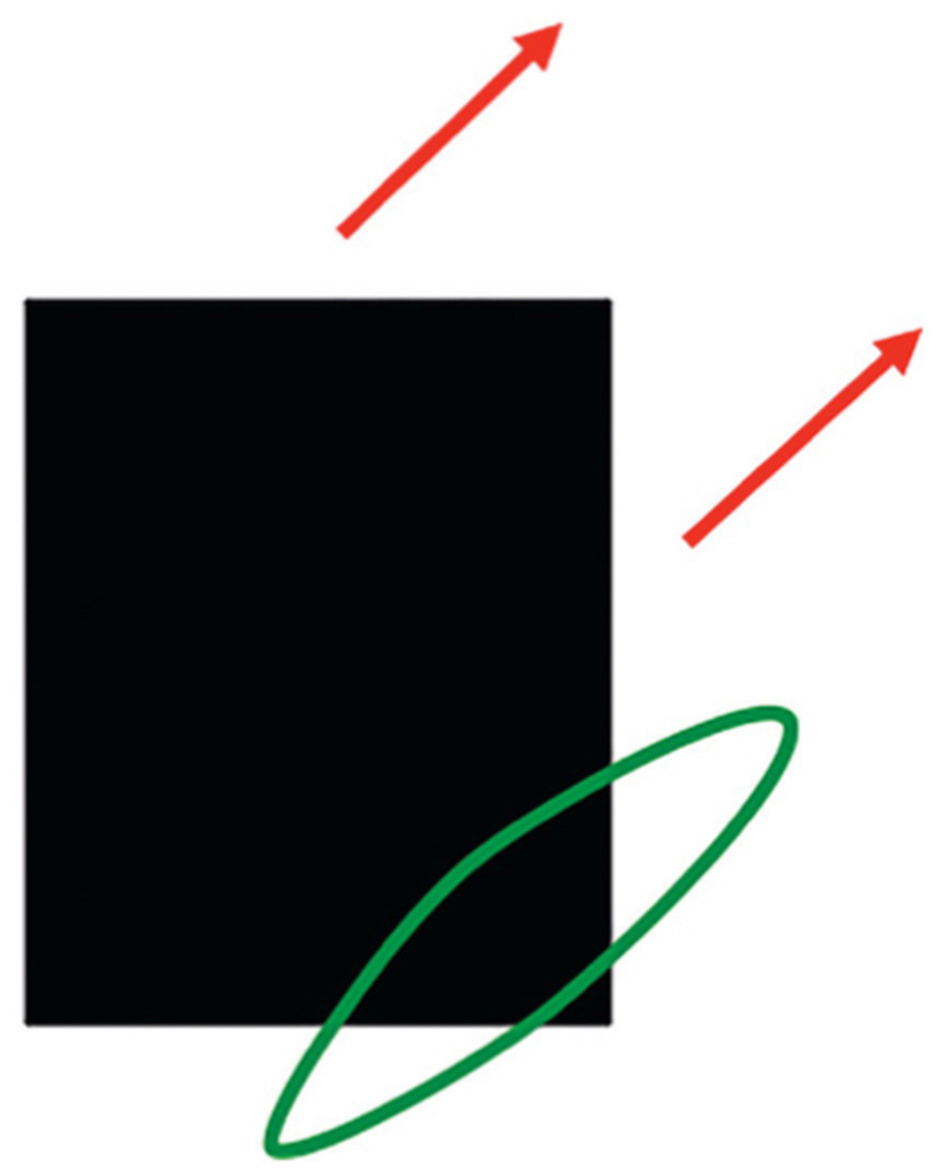
A long-range directional filter (oriented green ellipse) pools all possible contrast-sensitive sources of information, including oriented contrast changes at edges, that are moving in that direction. This estimate pools motion signals across all orientations and with all contrast polarities that move in the same direction through time. [Reprinted with permission from Grossberg (2021b)].

As I noted above, interacting brain regions control eye movements that maintain foveation on mommy as she moves. Suppose that the linear motion of mommy's body activates a long-range directional filter. Such a filter has an elongated shape that adds inputs from an object's motion signals over time that move in its preferred direction when they cross its receptive field (Figure 12). Arrays of such filters that are tuned to different preferred directions occur in the Where cortical stream (Albright, 1984; Rodman and Albright, 1987), where they compete across direction at each position to choose the filter that is most activated by the mommy's movement (Chey et al., 1997, 1998).

When a directional filter is activated for a sufficiently long time, its output signals trigger learning of a directional motion category in the Where stream (Farivar, 2009), which can learn an association with a descriptive word or phrase in the What stream, such as “left.”

After all the perceptual categories for recognizing “mommy,” “walks,” and “left” are learned, they can be associated with a linguistic phrase such as “mommy walks left” that is spoken by an adult speaker. Mechanistic details of how the ordered sequence of these words stored and learned are described below.

### Learning to say “mommy throws the ball” while observing her do so

How does a baby or child learn to say “Mommy throws ball” while observing mommy doing that? The first part of the sentence, “Mommy throws,” can be understood in much the same way as “Mommy walks.” In addition, when the child sees mommy pull her arm back before thrusting it forward, extreme arm position may be sufficient to learn a category for “throw” in the What stream, while the motion of the throw can be categorized in the Where stream.

As mommy completes the throw, the ball leaves her hand and continues moving in the same direction. Attention can then flow via a G-wave from mommy's face to her arm, and then to the ball. Perceptual categories that correspond to the events mommy, throws, and ball are activated and stored in a perceptual working memory in their correct temporal order, leading to learning of a perceptual sequence category, or list chunk. A heard sentence category of “mommy throws ball” can simultaneously be stored in a linguistic working memory, and trigger learning of its own list chunk. The linguistic list chunk learns an association with the perceptual list chunk, and conversely. The list chunks also send learned top-down signals to the working memory patterns that they categorize, which can then be performed from working memory in the correct order when a volitional GO signal from the basal ganglia turns on. After learning, seeing this event sequence can elicit a descriptive sentence.

### Where are nouns and verbs stored in the brain? Semantics and syntactics

What parts of the brain are used to store and understand a sentence such as “Watch mommy throw the ball”? The verbs “watch” and “throw” have cortical representations in the Where cortical stream. Nouns such as “mommy” and “ball” have cortical representations in the What cortical stream. Both noun and verb word representations are stored in the temporal and prefrontal cortices individually or in sequences.

Thus, understanding the meaning of a sentence such as “Watch mommy throw the ball” requires switching between the noun and verb representations in the What and Where cortical streams, respectively. Words such as the article “the” that help to structure sentences are part of *syntactics*, whereas the branch of linguistics that is concerned with meaning is called semantics. Traditional semantic studies do not link language utterances to their perceptual and affective meanings, e.g., Jackendoff (2006).

### How are item sequences stored in working memory and learned as list chunks?

All the sentences about mommy that are described above, indeed all the sentences in a language, are first stored in a working memory before they are learned. Before individual items in a sequence are stored, they are learned as item chunks that respond selectively when the distributed features that the item represents are presented. Phonemes and musical notes are examples of item chunks.

Sufficiently short sequences of item chunks can be temporarily stored in working memory. If such a sequence, say a short sentence, is stored frequently enough, it can be learned as a unitized, or compressed, list chunk. A list chunk selectively responds to prescribed sequences of item chunks that are stored in working memory. These processes occur in brain regions such as the ventrolateral prefrontal cortex (VLPFC) and the dorsolateral prefrontal cortex (DLPFC). The list chunks in these brain regions interact with other brain regions, including perirhinal cortex (PRC), parahippocampal cortex (PHC), amygdala (AMYG), lateral hypothalamus (LH), hippocampus (HIPPO), and the basal ganglia (BG). These interactions can choose predictions and actions that are most likely to succeed based on the sequential context of previously rewarded experiences. Figure 8 summarizes a macrocircuit of the *predictive adaptive resonance theory*, or pART, model of the cognitive and cognitive-emotional dynamics that model how these interactions work (Grossberg, 2018). pART includes neural models of seven prefrontal regions that interact to store, learn, and plan event sequences. These regions are colored green in Figure 8.

### Item and Order and Item-Order-Rank working memories

I introduced a universal model of working memory in 1978 and incrementally developed it with my collaborators to the present time (e.g., Bradski et al., 1994; Grossberg, 1978a,b, 2018, 2022; Grossberg and Pearson, 2008; Silver et al., 2011). I call it a “universal” model of working memory because it can be derived from a couple of simple hypotheses, and the same canonical circuit design, suitably specialized, can store auditory, linguistic, spatial, or motor sequences in multiple working memories that operate in parallel in the prefrontal cortex.

**Figure 13:**
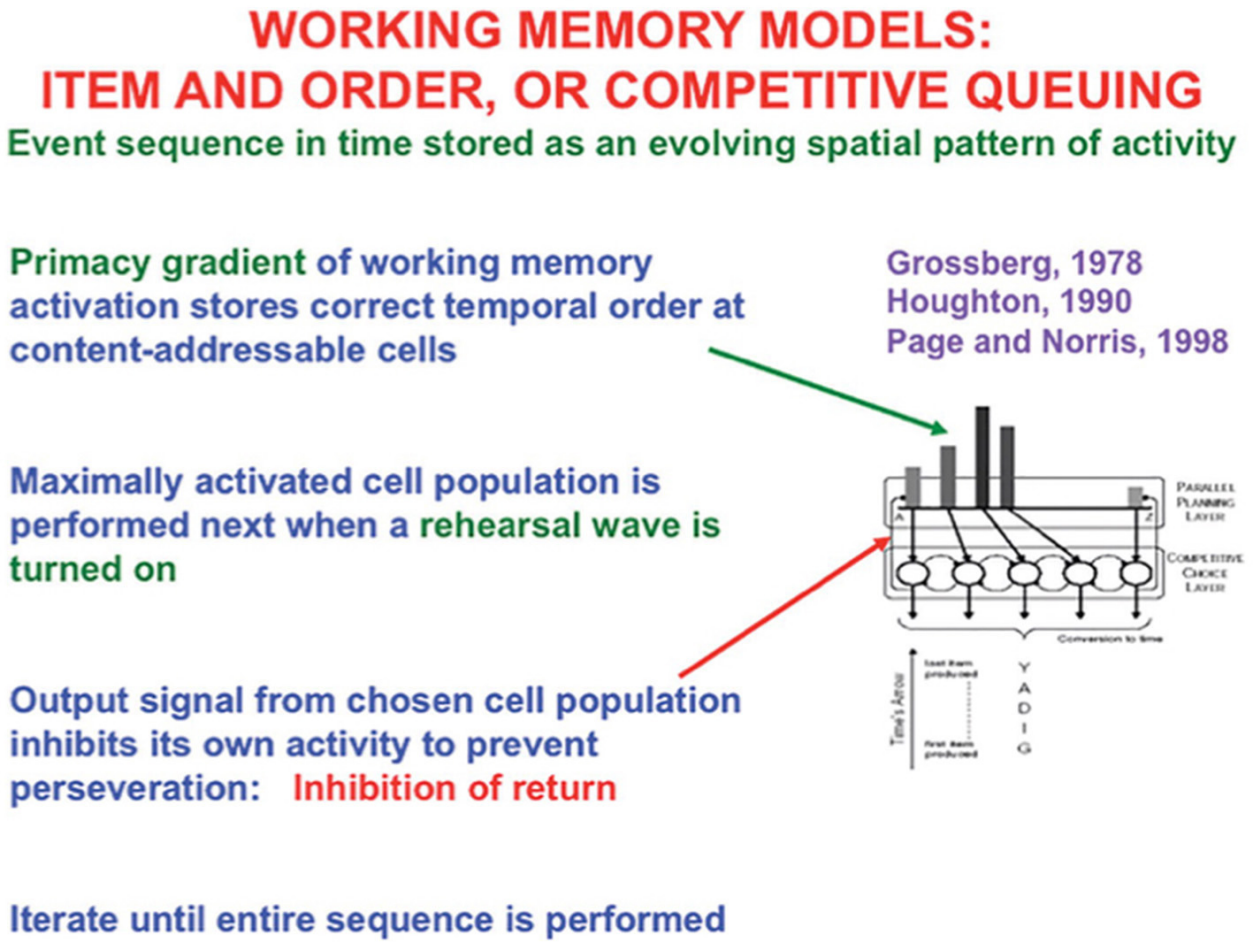
Item-and-Order working memory model. Item-and-Order working memory models explain data about free recall, during which subjects repeat list items that they have heard once in the order that they come to mind, as well as many other psychological and neurobiological data. Such a working memory model simulates how temporal series of events are stored as evolving spatial patterns of activity at content-addressible item categories. The categories with the largest activities are rehearsed first and self-inhibit their activity as they do so to prevent them from being rehearsed repeatedly or perseveratively. The laws whereby sequences of items are temporarily stored in working memory obey simple hypotheses concerning how list categories, or chunks, of sequences of stored items can be learned and stably remembered. [Reprinted with permission from Grossberg (2021b)].

The simplest model is called the Item-and-Order working memory because a sequence of inputs that occur one at a time is stored as an evolving spatial pattern of activation of item chunks that code the cell populations of the working memory (Figure 13). Individual cell populations thus represent list *items* and their *temporal order* of occurrence is stored by their relative activities within an activity gradient across the populations.

An Item-and-Order working memory cannot store a sequence in which some items are repeated, such as repeated letters in the sequence “ABACBD,” or repeated words in the lyric “my true love is true.” A generalization of this model, called the Item-Order-Rank, or IOR, model, can store sequences with repeats. Other IOR working memories can store the turns and distances traveled during navigation to a goal, the arm movements made during a dance, or the notes played in a musical melody.

### Why are IOR working memories unique? LTM Invariance Principle and stable chunking

Two kinds of evidence support the existence of IOR working memories in our brains (Grossberg, 2021b, 2022). First, they provide unified and principled explanations of many psychological and neurobiological data about working memory and list chunk dynamics. Second, they explain why and how sequences of items and events that are stored in working memory are learned and stably remembered through time as list chunks. In fact, Item-and-Order working memories can be derived from two simple postulates that enable their list chunks to be learned and stably remembered: the *LTM Invariance Principle* and the *Normalization Rule*. These postulates were used to derive mathematical equations for Item-and-Order working memories when I introduced them in Grossberg (1978a,b).

The LTM Invariance Principle prevents storage of longer lists of events in working memory (such as MYSELF) from causing catastrophic forgetting of previously learned list chunks of its shorter sublists (such as MY, SELF, and ELF). It guarantees that, if bottom-up inputs store a word in working memory and learn its list chunk, say for the word MY, then also storing the word SELF to complete storage and learning of the novel word MYSELF will not cause forgetting of the learned weights that activated the list chunk of MY.

The Normalization Rule just says that the maximum total activity that is stored across a working memory is independent of the number of activated cells. This rule follows from the fact that the cells in an Item-and-Order working memory *compete* among themselves via a recurrent shunting on-center off-surround network. Such networks occur ubiquitously in our brains because they solve what I call the *noise-saturation dilemma* (Grossberg, 1973, 2021b), which is solved by all cellular networks because it enables cells to store the relative sizes, and thus importance, of inputs in their activities without flattening the pattern of activity by saturating when inputs are too large, or distorting them in cellular noise when inputs are too small.

The LTM Invariance Principle and Normalization Rule imply that only short lists can be stored in working memory in a way that enables their performance in the correct temporal order. That is because a sufficiently short list can be stored as a *primacy gradient* of activity whose items can be recalled in the correct temporal order. In a primacy gradient, the first sequence item is stored with the most activity, the second item is stored with the next largest activity, and so on, until all items are stored (Figure 13). For example, the primacy gradient that stores the sequence “A-B-C” of items stores “A” with the highest activity, “B” with the second highest activity, and “C” with the least activity.

A stored spatial pattern in working memory is recalled as a temporal sequence of items when a rehearsal wave, or GO signal, from the basal ganglia uniformly activates all the working memory cells (Figure 13). The cell population with the highest activity is read out fastest because it exceeds its output threshold fastest. As it is read out, it self-inhibits its working memory activity via a recurrent inhibitory interneuron (Figure 13), a process that is often called the inhibition-of-return (Posner et al., 1985). Then, the cell population with the next largest activity can be read out, and so on, until the entire sequence is performed. Just three interacting processing levels are sufficient to store, learn, and perform long sequences of items or events that include repeats, such as in the lyric “our true love was true.” Grossberg (2022) illustrates how these circuits enable the learning and performance of musical lyrics and melodies (Figure 2).

Our brains do not need, nor do they have, many processing levels to store, learn, and perform sequential behaviors, in contrast to deep learning models that may need more than one hundred networks in a hierarchy, each with similar connectivity (Srivastava et al., 2015).

### Learning to use definite and indefinite articles in sentences

English language meanings cannot be fully understood without the indefinite and definite articles. The following quote from Study.com explains this distinction:

“An article is a word used to modify a noun, which is a person, place, object, or idea. Technically, an article is an adjective, which is any word that modifies a noun. Usually adjectives modify nouns through description, but articles are used instead to point out or refer to nouns. There are two different types of articles that we use in writing and conversation to point out or refer to a noun or group of nouns: definite and indefinite articles.”

“The definite article (the) is used before a noun to indicate that the identity of the noun is known to the reader. The indefinite article (a, an) is used before a noun that is general or when its identity is not known. There are certain situations in which a noun takes no article.”

For example, consider the meanings of the indefinite article “a” and the definite article “the” in the phrases “a ball” and “the ball.” The sentence “It is a ball” can refer to any ball, whereas the sentence “Watch the ball” refers to a particular ball.

### Combining definite and indefinite articles with nouns and verbs

Consider the sentences: “It is the ball” or “That is the ball” vs. “It is a ball” or “That is a ball.” Or the sentences “Watch the ball” vs. “Watch a ball.” The word “is” can precede a noun (object word) or a verb (action word). For example, the phrase “is a” disambiguates “is” to precede a noun, whereas “is throwing” illustrates how “is” can precede a verb.

An observer can say that “Mommy is throwing the ball” or “Mommy is throwing a ball” depending on whether a particular ball is intended. How does a baby learn the different meanings of “Mommy throws a ball” and “Mommy is throwing a ball”? Or of “Mommy throws the ball” and “Mommy is throwing the ball”? Both kinds of sentences refer to the same action, but replacing “throws” with “is throwing” emphasizes that the action is occurring and can be learned from a teacher while witnessing the throw as it happens.

The choice of articles “a” or “the” in such sequences also depends on whether they are in response to heard speech that is uttered by someone else, as in a sentence such as “Watch mommy throw the ball,” or self-generated speech in response to an externally viewed, or internally remembered, perceptual experience such as “Mommy threw a ball.” Since children learn their first languages by listening to teachers who know the language, the choice of article will depend on the perceptual experiences to which the teachers' utterances correspond.

### Attentional blocking and unblocking: how children learn to separate articles from nouns

Phrases such as ”a ball” or “the ball” can initially be learned as list chunks, or unitized representations, as a child listens to mommy speak about a perceptual event that involves a ball. How are these articles dissociated from the particular nouns with which they co-occur, so that the child can learn separate linguistic categories for articles and nouns, and thereby link the linguistic categories of nouns, such as “ball,” to a perceptual category of a/the ball.

Processes such as attentional blocking and unblocking clarify how this happens (Grossberg, 1975, 2018; Grossberg and Levine, 1987; Grossberg and Merrill, 1992, 1996; Grossberg and Schmajuk, 1989; Kamin, 1968, 1969; Pavlov, 1927). Attentional blocking of a word or perceptual object can occur when it is predictively irrelevant. It is then suppressed and not attended. Unblocking of a suppressed word or object can occur when it becomes predictively relevant again.

Since the word “ball” is always associated with the perceptual experience of a ball, it predictively occurs in phrases such as “a ball” and “the ball.” However, the articles “a” and “the” are not, because they can co-occur with many other words and are chosen via a one-to-many mapping from each article to the many words with which it co-occurs in sentences. When the articles are suppressed by blocking, the primacy gradient that stores the word “ball” in working memory can trigger learning of a linguistic category of the word that can be associated with visual categories of the perceptual experiences of seeing a/the ball.

An article can remain predictively irrelevant and blocked until a predictive perceptual context, and thus a language meaning, is associated with a phrase such as “a ball,” when an unfamiliar ball is experienced, or “the ball” when the ball is a particular or familiar one. In these situations, the phrases “a ball” and “the ball” in working memory may trigger learning of their own list chunks.

Behavioral interactions between a teacher and a learner, like the following ones, may help to understand how the meanings of these phrases are learned: Suppose that a child says “Mommy throws ball,” and mommy says in return “This is the ball daddy bought.” If experiences like this happen enough, the child can learn that “the ball” may refer to a particular or familiar ball and, as noted above, the phrase “the ball” may be learned as a list chunk in response to its recurring representation as a primacy gradient in working memory.

Definite and indefinite articles contribute to meaning by interacting with both perceptual and cognitive processes: Choosing which article “a” or “the” to store in working memory depends on perceiving, or imagining, the object that the article modifies. The article “the” may refer to a specific or familiar ball, as in the sentence: “Mommy threw the ball.” The article “a” may refer to any ball, including an unfamiliar one, as in the sentence: “Pick a ball from the basket.” With this perceptual information available, articles are inserted into phrases and sentences that are stored in a linguistic working memory, along with the nouns that they modify. The stored item sequence can then be performed in response to a volitional GO signal.

Adjectives and adverbs can influence what is perceived when constructing a sentence, or imagined when hearing the sentence, e.g., “big ball,” “quickly running,” etc. Hearing adjective-noun and adverb-verb phrases can also trigger perceptual memories of such experiences. These words can be inserted in sentences in much the same way as articles are.

### Learning to associate visual objects with auditory names

Where and how an article such as “a” or “the” is inserted in the brain into a phrase or sentence is clarified by where visual events like objects are unitized through learning into object categories that are then associated with their learned auditory names in working memory. The perceptual meaning of a noun's name–e.g., “ball”-emerges from being associated through bi-directional visual-auditory learning with a learned visual category of the ball. The ability to recognize an object as a ball may not determine whether the name “ball” is modified by the article “a” (“That's a ball”) or “the” (“That's the only ball that I own”). Using “the” could occur when a combination of the ball's features is familiar, such as its size, markings, texture, or color, or because it is used in a definite context, e.g., “Watch mommy throw the ball.”

Multiple brain processing stages are used in either case, starting with conscious seeing of the ball. As noted above, the functional units of 3D vision are perceptual boundaries and surfaces, which are computed in the striate and prestriate visual cortices, including cortical areas V1, V2, and V4, in the What, or ventral, cortical stream (Gegenfurtner, 2003; Motter, 1993; Sereno et al., 1995). After the processing of visual boundaries and surfaces is complete, they are then learned as perceptual categories in the inferotemporal cortex. As summarized above, a particular view of surface, such as mommy's face, can be learned and recognized by a category in the posterior inferotemporal cortex, or ITp. An invariant category that selectively responds to multiple views, positions, and sizes of mommy's face can be learned within the anterior inferotemporal cortex, or ITa. Such an invariant category may reciprocally interact via bi-directional adaptive connections with all the view categories of mommy's face in ITp. If the linguistic phrase “mommy's face” activates the invariant category that represents it in ITa, all the view-specific categories in ITp can then also be primed by top-down signals. As noted above, this interaction enables joint attention to occur between where mommy is looking and when her child will look as well.

These visual object recognition categories, in turn, activate additional processes at higher cortical areas, such as those that code familiarity about objects, including anterior temporal cortex, anterior occipitotemporal sulcus, anterior fusiform gyrus, posterior superior temporal sulcus, and the precentral gyrus over the frontal cortex (Bar et al., 2001; Bonner and Price, 2013; Chao et al., 1999; Haxby et al., 2001; Huth et al., 2012; Kovács, 2020; Ramon and Gobbini, 2018; Rajimehr et al., 2009; Sugiura et al., 2011). Auditory object name categories and facts about these objects may be computed in the anterior temporal cortex, among other cortical areas (Bemis and Pylkkänen, 2013; Hamberger et al., 2005).

### Many-to-one and one-to-many associative maps

**Figure 14:**
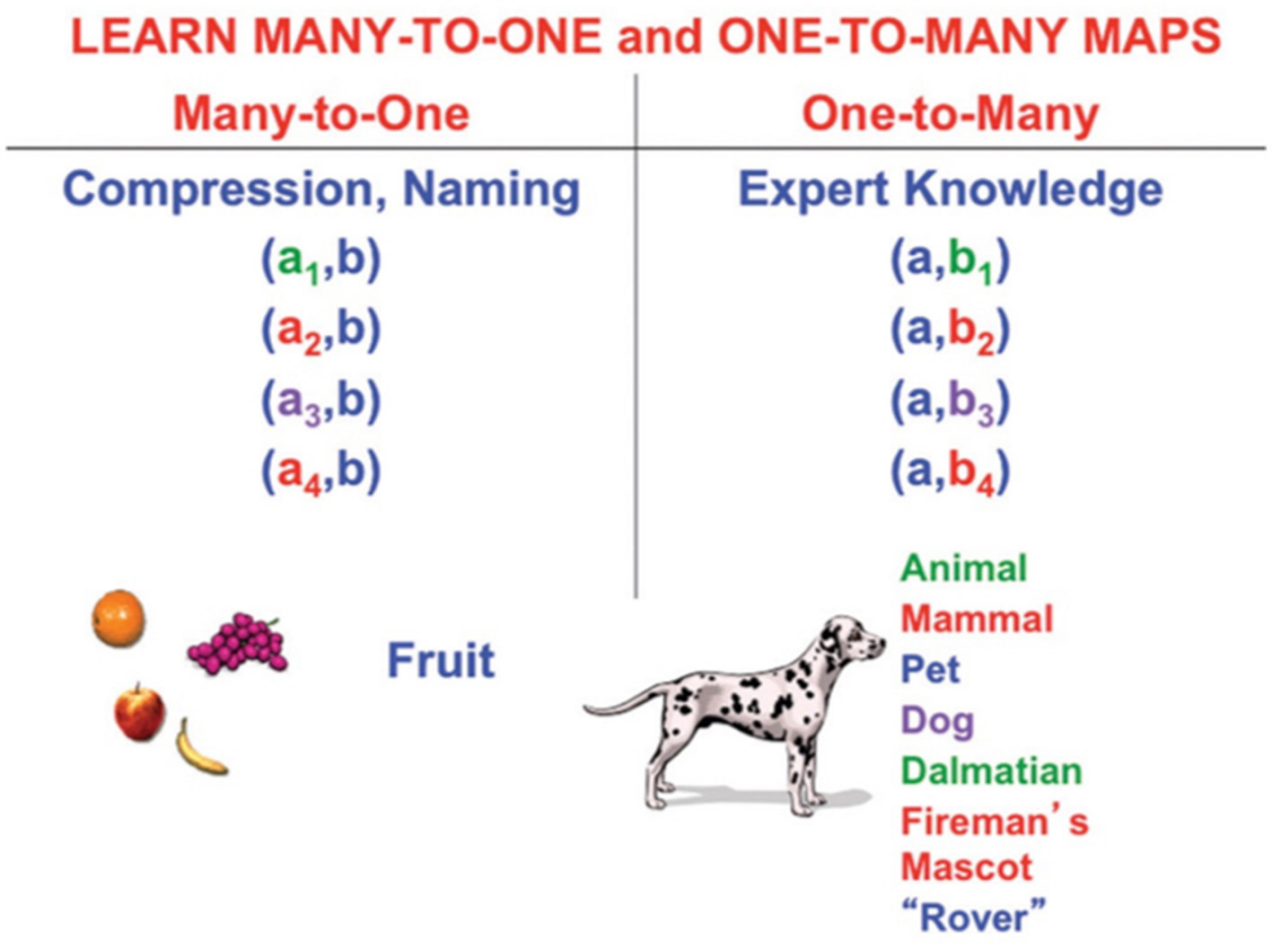
Many-to-one and one-to-many maps. Humans and other terrestrial animals, no less than autonomous adaptive intelligent agents, need to be able to learn both many-to-one and one-to-many maps. See the text for details. [Reprinted with permission from Grossberg (2021b)].

Visual recognition categories and auditory name categories can be linked through learning by an associative map. Figure 14 depicts two kinds of associative maps: many-to-one maps and one-to-many maps. In Figure 14, a many-to-one map maps visual images of multiple different kinds of fruit into the same name “fruit.” The one-to-many map in Figure 14 associates the image of a dog with many different words to describe it, ranging from the general words such as “animal” to the specific name of a particular dog “Rover.”

**Figure 15:**
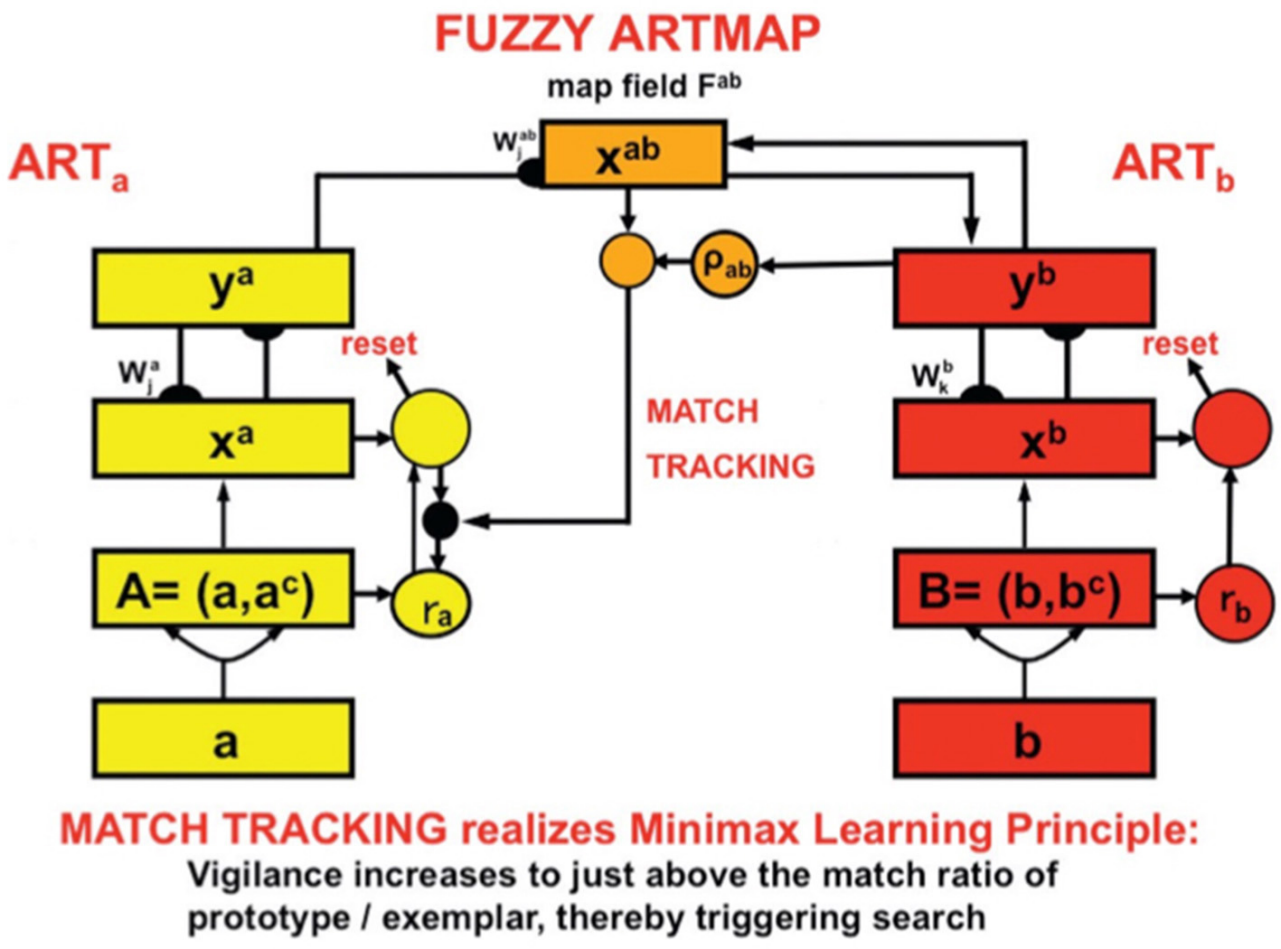
A system such as Fuzzy ARTMAP can learn to associate learned categories in one ART network (ARTa) with learned categories in a second ART network (ARTb) via a Map Field. Because both bottom-up and top-down interactions occur in both networks, a bottom-up input pattern to ARTa can learn to generate a top-down output pattern from ARTb, and thereby learn a prediction from one type of information (e.g., visually experienced printed and written fonts of a given letter of the alphabet) to another type of information (e.g., the auditory name of the letter), or from a prescribed combination of medical symptoms, tests, and treatments to a prediction of the length of stay in the hospital of the treated patient. [Reprinted with permission from Grossberg (2021b)].

Figure 14 illustrates how learning of a many-to-one map uses two stages of learning: first, multiple visual fonts of a letter A trigger learning of multiple visual categories that selectively respond to variations of each letter font. Multiple categories emerge because the fonts are defined by different visual features. Next, these visual categories are all associated with the same auditory name of the letter via a Map Field (Figure 15).

Learning a many-to-one map can be done by quite a few associative learning models. However, learning a one-to-many map requires a model such as ARTMAP, for learning binary mappings, or fuzzy ARTMAP for learning binary or analog mappings (Asfour et al., 1993; Bradski and Grossberg, 1995; Carpenter, 1997, 2003; Carpenter et al., 1992, 1991; Carpenter and Tan, 1995; Carpenter et al., 1997, 1998, 2005; Carpenter and Ravindran, 2008; Granger et al., 2000; Grossberg and Williamson, 1999). ARTMAP is needed because, after learning, say, that a dog image is associated with “animal,” when associating the dog image also with “Rover,” learning the “Rover” association can erase the “animal” association in many models, including back propagation and deep learning. ARTMAP models dynamically buffer the memories of previous associations, while also driving a memory search that will discover, focus attention on, and learn a new category to represent, the particular combination of critical visual features that distinguish “Rover” from other dogs.

### Map fields are working memories

The discussions above have clarified how observing mommy throwing a ball can initiate storage of this sequence of events in a linguistic working memory as a descriptive sequence of words, as in the sentence “mommy throws the ball.” Putting together the discussions of working memories and Map Fields leads to the conclusion that a Map Field can also serve as a working memory in which linguistic sequences can be stored in response to sequential activation of their visual categories through time. A variation of this design is one wherein a Map Field topographically inputs to a working memory, but the Map Field itself does not have the recurrent interactions or volitional GO signal modulation of working memory.

### Adaptive resonance between bottom-up adaptive filters and top-down learned expectations

Figure 14 shows only bottom-up adaptive pathways between the distributed feature pattern of each letter and its visual category. In the brain, as well as in Adaptive Resonance Theory, or ART, models of object category learning, there are both bottom-up and top-down adaptive pathways, as in the ARTa and ARTb models in the Fuzzy ARTMAP architecture of Figure 15. The bottom-up pathways form an adaptive filter whose pathways end with adaptive weights, or long-term memory (LTM) traces, that are depicted by hemidisks in Figure 15. These adaptive weights learn the critical feature patterns that control ARTMAP predictions. The top-down pathways embody expectations that learn critical feature patterns and focus attention on them. Critical feature patterns include only the feature combinations that past learning has shown to control learning and correct predictions. Outlier features are suppressed during learning because they are predictively irrelevant.

When both bottom-up and top-down pathways are simultaneously active, the activity patterns that they select synchronize, amplify, and focus attention on the critical feature pattern that reliably codes the correct category. It is the synchronous and sufficiently sustained resonance between features and categories that triggers fast learning within the bottom-up and top-down adaptive weights that lead to and from the currently active category. That is why I call the resonance an *adaptive* resonance. During such a resonance, top-down matching by a learned expectation protects the learned adaptive weights from being destabilized by catastrophic forgetting, thereby solving the *stability-plasticity dilemma*: they support fast learning (plasticity) while dynamically buffering the learned weights from experiencing catastrophic forgetting [stability; see Grossberg (2021b) for further details].

Learning of adaptive resonances takes place within what I have called the *attentional system* (Figure 1). When input patterns do not match currently active learned top-down expectations well enough, this mismatch is too novel, or surprising, to be incorporated into those categories. This mismatch activates a computationally complementary *orienting system*, which triggers directed search, or hypothesis testing, in the attentional system, leading either to discovery of an already learned category that provides an adequate match, or activation of uncommitted category cells to learn the novel category. The *free-energy principle* of Friston (2010) also incorporates a role for surprise in its learning process.

## Concluding remarks: human and machine learning of large language models with meaning

This article advances the analysis by Grossberg (2023) of how children and other students can learn small numbers of language utterances that have perceptual and affective meanings. The article makes this advance by explaining how humans, and neural network models of their brain dynamics, learn to consciously perceive and recognize an unlimited number of visual scenes. Then, bi-directional associative links can also be learned and stably remembered between any number of scenes and descriptive language utterances of them, as well as the emotions that these scenes evoke. Adaptive Resonance Theory circuits control the learning and the self-stabilizing memories of these processes. The article also surveys many of the neural models that are needed to carry out this goal, and compares them with models of other authors. Taken together, these models provide a blueprint for realizing Autonomous Adaptive Intelligence and Artificial General Intelligence.

I ended the exposition in Grossberg (2023) by quoting Ludwig Wittgenstein from his classic *Tractatus Logigo-Philosophicus* (Wittgenstein, 1922) in which Wittgenstein noted that “the limits of my language mean the limits of my world.” The current article greatly expands the language utterances and their perceptual and affective meanings with which to come closer to “the limits of my language.” As I noted in the study by Grossberg (2023), it will require many scientists working for many years to model all the language utterances and their meanings that we can express about our expanding experiences in the world throughout our lives. If the resources of Google DeepMind that have funded applications of Deep Learning and LLMs could also be directed to this goal, its realization will be greatly accelerated.

## Data Availability

The original contributions presented in the study are included in the article/supplementary material, further inquiries can be directed to the corresponding author.
